# Expanding the Biological Role of Lipo-Chitooligosaccharides and Chitooligosaccharides in *Laccaria bicolor* Growth and Development

**DOI:** 10.3389/ffunb.2022.808578

**Published:** 2022-02-14

**Authors:** Manuel I. Villalobos Solis, Nancy L. Engle, Margaret K. Spangler, Sylvain Cottaz, Sébastien Fort, Junko Maeda, Jean-Michel Ané, Timothy J. Tschaplinski, Jesse L. Labbé, Robert L. Hettich, Paul E. Abraham, Tomás A. Rush

**Affiliations:** ^1^Bioscience Division, Oak Ridge National Laboratory, Oak Ridge, TN, United States; ^2^Graduate School of Genome Science and Technology, University of Tennessee, Knoxville, Knoxville, TN, United States; ^3^Université Grenoble Alpes, CNRS, CERMAV, Grenoble, France; ^4^Department of Bacteriology, University of Wisconsin-Madison, Madison, WI, United States; ^5^Department of Agronomy, University of Wisconsin-Madison, Madison, WI, United States

**Keywords:** *Laccaria bicolor*, lipo-chitooligosaccharides, polarized growth, proteomics, chitooligosaccharides

## Abstract

The role of lipo-chitooligosaccharides (LCOs) as signaling molecules that mediate the establishment of symbiotic relationships between fungi and plants is being redefined. New evidence suggests that the production of these molecular signals may be more of a common trait in fungi than what was previously thought. LCOs affect different aspects of growth and development in fungi. For the ectomycorrhizal forming fungi, *Laccaria bicolor*, the production and effects of LCOs have always been studied with a symbiotic plant partner; however, there is still no scientific evidence describing the effects that these molecules have on this organism. Here, we explored the physiological, molecular, and metabolomic changes in *L. bicolor* when grown in the presence of exogenous sulfated and non-sulfated LCOs, as well as the chitooligomers, chitotetraose (CO4), and chitooctaose (CO8). Physiological data from 21 days post-induction showed reduced fungal growth in response to CO and LCO treatments compared to solvent controls. The underlying molecular changes were interrogated by proteomics, which revealed substantial alterations to biological processes related to growth and development. Moreover, metabolite data showed that LCOs and COs caused a downregulation of organic acids, sugars, and fatty acids. At the same time, exposure to LCOs resulted in the overproduction of lactic acid in *L. bicolor*. Altogether, these results suggest that these signals might be fungistatic compounds and contribute to current research efforts investigating the emerging impacts of these molecules on fungal growth and development.

## Introduction

Mycorrhizal associations are important mutualisms between plant roots and fungi that allow plants to acquire water and nutrients from the environment in exchange for photosynthates (Jeffries et al., 2003; Bonfante and Anca, 2009; Plett and Martin, 2018; Tedersoo et al., 2020). In the past years, there has been a continuous effort to advance the understanding of the molecular signaling mechanisms used by fungi to colonize plants in both natural and agricultural environments (Bonfante and Genre, 2010; Pan et al., 2013; Kamel et al., 2017; Maclean et al., 2017; Choi et al., 2018). An example is the production of lipo-chitooligosaccharides (LCOs—also known as Nod factors) that arbuscular mycorrhizal (AM) and ectomycorrhizal fungi (ECM) produce to stimulate colonization of host plant roots (Maillet et al., 2011; Cope et al., 2019; Khokhani et al., 2021). The production of LCOs was also shown to cause diverse effects on a variety of plants with or without mycorrhizal fungi (Tanaka et al., 2015).

LCOs were first discovered and characterized in rhizobia bacteria (Lerouge et al., 1990; Dénarié et al., 1996; Poinsot et al., 2016) and later found to be produced by most fungi (Maillet et al., 2011; Cope et al., 2019; Rush et al., 2020). These amphiphilic molecules are polymers made of three to five N-acetyl glucosamine (GlcNAc) residues with β-(1,4) linkages modified with a long-chain fatty acyl group and various other functional groups (Lerouge et al., 1990; Dénarié et al., 1996; Malkov et al., 2016). Most fungi produce sulfated LCOs (sLCOs) or non-sulfated LCOs (nsLCOs) with a palmitic (C16:0) or oleic (C18:1) acid (fatty acid chain) attached to the first chitin monomer and have either a chitotetraose, tetra-N-acetyl (CO4), or chitopentaose, penta-N-acetyl (CO5) backbone (Rush et al., 2020). CO4 and CO5 are *N-*acetyl chitooligosaccharides and should not be confused with chitosan oligomers (Yin et al., 2016). Other chemical substitutions have been identified in fungal LCOs, but their role has not yet been determined (Rush et al., 2020). LCOs and COs are perceived on the surface of the root cells of host plants by a group of lysine-motif receptor-like kinases (LysM) (Buendia et al., 2018) which in return activate a central plant signaling cascade known as the Common Symbiosis Pathway (CSP) (Feng et al., 2019; Cope et al., 2021; Wu et al., 2021). The immediate response of plants to the perception of LCOs or COs, produced by symbiotic or beneficial endophytic fungi, are oscillations in nuclear calcium (Ca^+2^) concentration levels in the epidermal cells of host roots, which subsequently cause Ca^+2^/calmodulin-dependent protein kinases to regulate the activity of several transcription factors necessary for the establishment of symbiosis or mutualistic associations (Chabaud et al., 2011; Genre et al., 2013; Buendia et al., 2016; Luginbuehl and Oldroyd, 2017; Choi et al., 2018; Cope et al., 2019, 2021; Feng et al., 2019; Skiada et al., 2020). It remains unknown if LCOs and COs from saprotrophic and pathogenic fungi trigger the CSP and if they could use this signaling pathway for colonization of a host plant.

In addition to their impact on their host plants, LCOs and COs can alter fungal physiology and transcriptomics. The observed fungal behavior may be attributed to sensing diffusible chemical signals (Aleklett and Boddy, 2021). For example, in the saprotrophic and opportunistic human pathogen, *Aspergillus fumigatus*, growth with C16:0 sulfated LCOs (C16:0 sLCOs) resulted in differential expression of genes encoding proteins associated with cell membrane activities and cell wall processes that led to significantly reduced hyphal branching (Rush et al., 2020). Also, C16:0 sLCOs were shown to influence fungal behavior in other opportunistic human pathogens like increased pseudohyphae formation in *Candida glabrata* and increased cell proliferation in *Rhodotorula mucilaginosa* (Rush et al., 2020). When a fungus is co-inoculated with a host plant, LCOs were shown to increase disease incidence of Sclerotinia stem rot on susceptible lines of soybeans (Marburger et al., 2018), increased colonization from arbuscular mycorrhizal fungi in legumes (Maillet et al., 2011), and increased mantle width and Hartig net formations in *L. bicolor* colonizing poplar roots (Cope et al., 2019). Although few investigations reported the role of LCOs on fungal development or their influence on co-colonization within a host, the recent onset of evidence is pointing toward alternative roles of LCOs and COs outside of symbiosis. These molecules were reported to be produced by fungi on substrate media in the absence of a host to potentially influence the development of the producing organism (Rush et al., 2020). However, the mechanisms by which these molecules are triggering these physiological changes remained largely unknown. Therefore, the goal of this study is to provide a baseline cognizance of the structure, function, and regulation of fungal behavior caused by LCOs and COs.

Ectomycorrhizal fungi, such as *Laccaria bicolor*, play a crucial role in various forest ecosystems. They provide a nutritional benefit to their host plants and help mitigate the impact of a wide range of biotic and abiotic stresses (Martin et al., 2008). Given the recognition of LCOs and COs as critical signaling molecules in regulating plant and fungal behaviors, we set to study these signals' influence on *L. bicolor* physiology, protein abundance changes, and metabolite production. We hypothesize that when the fungus is living within a soil microbiome, it uses specific types of LCOs or COs to regulate its development, regardless of its symbiosis with the host plant. Previously, different LCO structures were shown to increase lateral root development in poplar or improve colonization of *L. bicolor* with its poplar host (Cope et al., 2019). However, understanding how LCOs or COs influence *L. bicolor* development by itself has not been investigated. Therefore, we expose the fungus to the same sLCOs and nsLCOs purified from rhizobia, and to the same chitooligosaccharides chitotetraose (CO4) and chitooctaose (CO8) used by Cope et al. (2019). A key consideration for these experiments is that various LCO types are used for specific host symbiosis and phenotypic responses (D'Haeze and Holsters, 2002). This is noteworthy because as previously shown, sLCOs and nsLCOs extracted from rhizobia or numerous fungi were shown to cause root hair branching in *Medicago truncatula* and *Vicia sativa*, respectively (Cope et al., 2019; Rush et al., 2020), indicating that the structure of LCOs is potentially similar enough between rhizobia and fungi to elicit this phenotype in legumes. Therefore, for this manuscript, we assume that the sLCOs and nsLCOs used are not recognized as foreign signals, but commonly produced molecules by rhizobia and most fungi, including *L. bicolor*. Later, we tested the effect of synthetically made LCOs on *L. bicolor* to mimic the structure of LCOs abundantly produced by this fungus. We also examined the effects from CO4, one of the possible backbone molecules of an LCO (Lerouge et al., 1990), and CO8, an elicitor for plant defense responses, but not a backbone molecule of LCOs (Kuchitsu et al., 1997; Buendia et al., 2018; Feng et al., 2019). Lastly, since rhizobia produce LCOs to initiate symbiosis with legumes under field conditions (Kidaj et al., 2012; Siczek et al., 2014), presumably, *L. bicolor* will perform a similar function with poplar. Therefore, the findings from our *in vitro* studies would help understanding the influence of LCOs and COs on *L. bicolor* in field studies. Finally, a proteomic approach was subsequently used to provide molecular insights into the changes in *L. bicolor* protein abundance when growing with or without the various forms of LCOs and COs. Differential protein abundance underpinning reduced growth for specific LCOs and COs represent a substantial alteration in proteome expression in biological processes related to polarized growth.

## Materials and Methods

### Fungal Growth Experiments

Square Petri dishes with gridlines (Thomas Scientific) were filled with 50 ml of Pachlewski agar medium (P20). LCO treatments were synthesized resulting in LCO types: C16:0 sulfated (C16:0 sLCO), C16:0 non-sulfated (C16:0 nsLCOs), C18:1 sulfated (C18:1 sLCOs), and C18:1 non-sulfated (C18:1 nsLCOs) in 0.005% ethanol/water (v/v) as used before (Rush et al., 2020). COs were chitotetraose, tetra-N-acetyl (CO4) (IsoSep, Tullinge, Sweden—Product Number: 45/12-0050) and chitooctaose, octa-N-acetyl (CO8) (IsoSep, Tullinge, Sweden—Product Number: 57/12-0001) in 0.005% ethanol/water (v/v) (Rush et al., 2020). We used a concentration of 10^−8^ M because of its biological influence on fungi shown in previously studies (Maillet et al., 2011; Cope et al., 2019; Rush et al., 2020). Individually applied LCOs or COs treatments had a concentration of 10^−8^ M, were spread evenly across the agar medium with a sterile cell spreader and set to dry. The solvent control was 0.005% ethanol/water (v/v). A single fungal plug of *L. bicolor* was then cut with a sterile 1-cm^2^ area core borer and placed in the middle of the agar medium with the treatment. There were five biological replications per treatment. Inoculated plates were placed in a dark incubator at 25°C.

*Laccaria bicolor* strain S238N has radial hyphal growth patterns in culture (Labbe et al., 2014), therefore for the fungal growth data, diameter measurements were taken based on the cardinal points every odd day for 21 days post-inoculation (dpi). Growth area was measured as the area of fungal growth minus the area of the core borer. For the clamp connections data, there were five technical replications per biological replicate. Measurements were taken after 7, 15, and 21 dpi. The total number of clamp connections observed within a fixed area was counted per technical replication. A fixed area is all the clamp connections counted within the image taken. The average was used for the total number of clamp connections observed per biological replication. Hyphal branching measurements were taken 3 dpi, with five technical replicates per biological replicate. In addition, five random apical branches were counted for secondary branches within a technical replicate. Secondary branches were counted from 400 μm of the apical branch hyphae starting from the tip of the branch. The ratio is determined by the number of secondary branches counted within 400 μm of apical branch starting from the tip of the apical branch. The average of that ratio was used for the biological replication value. Statistical analyses were performed using GraphPad Prism software version 9.0.0 (GraphPad, San Diego, CA). Welch's one-way ANOVA was performed with an unpaired Welch's *t*-test per species, testing treatment group responses against solvent control responses.

### *Laccaria bicolor* Samples for Proteomics and Metabolomics Analysis

*Laccaria bicolor* strain S238N was grown for 21 days at 25°C from a single fungal plug cut with a sterile 1 cm^2^ area core borer. Fungal plugs were shaken at 200 rpm in 250 ml Erlenmeyer flasks filled with 50 ml of Pachlewski medium and inoculated with a concentration of 10^−8^ M of individual LCOs or COs. LCO treatments were mixtures of sLCOs or nsLCOs purified from *Sinorhizobium meliloti* and *Rhizobium* sp. IRBG74, respectively, and resuspended in 0.005% ethanol/water (v/v), as previously published (Maillet et al., 2011; Mukherjee and Ane, 2011; Sun et al., 2015; Cope et al., 2019). COs were chitotetraose, tetra-N-acetyl (CO4), and chitooctaose, octa-N-acetyl (CO8) in 0.005% ethanol/water (v/v) (Rush et al., 2020). Each treatment had individually applied applications of LCOs or COs with three biological replications for proteomics analysis, and four replications used for metabolomics analysis. In addition, fungi grown in media inoculated with 0.005% ethanol/water were used as solvent controls.

### Sample Preparation for LC-MS/MS Proteomics

*Laccaria bicolor* samples were suspended in 1 mL of SDS lysis buffer (2% sodium dodecyl sulfate in 100 mM NH_4_HCO_3_ solution). Samples were physically disrupted by ultrasonication using a pulse amplitude of 20% for 10 secs on and 10 secs off for a total of 2 min. Crude lysates were then boiled for 10 min at 95°C. Later, samples were centrifuged at 21,000 × g for 10 min to pre-clear the sample of DNA and other cellular debris and supernatants transferred to fresh Eppendorf tubes. Disulfide bond disruption was achieved by adjusting the pre-cleared protein extracts to 10 mM dithiothreitol and incubating the samples at 90°C for 10 mins. Cysteines were blocked by adjusting each sample to 30 mM iodoacetamide, followed by incubation in the dark for 15 min at room temperature. Proteins were precipitated using chloroform-methanol-water extraction. Dried protein pellets were resuspended in 250 μL of a 2% sodium deoxycholate (SDC) solution in 100 mM NH4HCO3, and protein amounts were estimated using a NanoDrop One^c^ spectrophotometer (Thermo Scientific). For each sample, aliquots of ~100 μg of protein, or all protein if less was obtained, were digested using sequencing-grade trypsin (Promega, 1:75 [wt/wt]) overnight under constant shaking at 37°C (600 rpm, Eppendorf Thermomixer). The second round of trypsin digestion was performed under the same conditions as before but for 3 h. Peptide mixtures were then adjusted to 0.5% formic acid to precipitate SDC. Hydrated ethyl acetate was added to each sample at a 1:1 (vol/vol) ratio three times to remove the SDC effectively. Samples were then placed in a SpeedVac concentrator (Thermo Fisher Scientific) to remove the ethyl acetate and further concentrate the sample. The peptide-enriched flow-through was quantified with the same NanoDrop One^C^ spectrophotometer as before.

### LC-MS/MS Analysis

Peptide samples were analyzed by automated one-dimensional LC-MS/MS analysis using a Vanquish ultra-HPLC (UHPLC) system plumbed directly in-line with a Q Exactive Plus mass spectrometer (Thermo Scientific) outfitted with a trapping column coupled to an in-house-pulled nanospray emitter. The trapping column (inner diameter, 100 μm) and the nanospray emitter (inner diameter, 75 μm) were packed with 5-μm Kinetex C18 reverse-phase resin (Phenomenex) to 10 and 30 cm, respectively. For each sample, peptides (2 μg) were loaded, desalted, separated, and analyzed across a 210-min organic gradient with the following parameters: sample injection followed by a 100% solvent A chase from 0 to 30 min (load and desalt), a linear gradient of 0–25% solvent B (70% acetonitrile, 30% water, and 0.1% formic acid) from 30 to 240 min (separation), a ramp to 75% solvent B from 240 to 250 min (wash), re-equilibration to 100% solvent A from 250 to 260 min, followed by maintaining 100% solvent A from 260 to 280 min. Eluting peptides were measured and sequenced by data-dependent acquisition with the Thermo Xcalibur v 4.2.47 software using the same parameters reported before (Johnson et al., 2017).

### Peptide Identification

MS raw data files were searched against the *Laccaria bicolor* Uniprot protein database (ID. UP000001194, downloaded on November 23, 2020), to which commonly contaminated proteins had been added using Proteome Discover v2.3 (Thermo Fischer Scientific, USA). Each MS/MS raw data file was processed with the SEQUEST HT database search algorithm, and confidence in peptide-to-spectrum (PSM) matching was evaluated by Percolator (Kall et al., 2007). SEQUEST HT was configured to derive fully tryptic peptides with the following parameters: max 2 missed cleavages, minimum peptide length of 6 amino acids, the maximum number of charge states of 4, a precursor mass tolerance of 10 ppm (ppm), a fragment mass tolerance of 0.02 Da, a static modification on cysteines (iodoacetamide; +57.0214 Da), and dynamic changes on methionine (oxidation; 15.9949). Peptides and PSMs were considered identified at *q* < 0.01, and proteins were required to have at least one unique peptide sequence. Functional annotations of proteins in the *L. bicolor* database were generated with the OmicsBox v1.2.4 software (BLASTp against non-redundant NCBI database of fungal sequences, E-value cutoff <1.0 E10^−3^; followed by InterPro search, GO Mapping, GO Annotation, and EggNOG Mapper with default parameters).

### Proteomics Data Analysis

Protein abundance values reported by Proteome Discoverer were log2-transformed, LOESS and mean-centered normalized across the entire dataset using the InfernoRDN software (Polpitiya et al., 2008). The normalized dataset was then imported to the Perseus software v.1.6.14.0 (Tyanova et al., 2016) for subsequent analysis. Relative quantification of proteins was limited to proteins with non-zero abundance values identified in at least two out of three biological replicates of at least one treatment condition (CO4, CO8, sLCOs, nsLCOs, Solvent Control). Abundance values for proteins with missing values were imputed with random values drawn from a normal distribution (width 0.3, downshift 2.8). ANOVA test (permutation-based FDR <0.05) was performed across all treatments, followed by *post-hoc* Tukey-HSD to determine significant treatment comparisons. Proteins were characterized as significantly differentially abundant if they passed the significance threshold of *p* ≤ 0.05 and absolute log2 fold-change difference ≥ 1.

### Metabolite Extraction and Analysis

Metabolites were extracted overnight from ~15 mg of lyophilized *L. bicolor* isolates with 2 ml of 80% ethanol to which sorbitol (50 μl of 1 mg/ml aqueous solution) was added as an internal standard. Following centrifugation, at 4,200 rpm and 4°C for 20 min, the extract was decanted, and overnight extraction and centrifugation were repeated with an additional 2 ml of 80% ethanol. The two extracts were combined, and a 1 ml aliquot was dried under a stream of nitrogen. The dried extracts were dissolved in 500 μl of silylation-grade acetonitrile followed by the addition of 500 μl of N-methyl-N-trimethylsilyltrifluoroacetamide (MSTFA) with 1% trimethylchlorosilane (TMCS) (Thermo Scientific, Bellefonte, PA) and heated for 1-hr at 70°C to generate trimethylsilyl (TMS) derivatives. After 1 day, a 1 μl aliquot was injected into an Agilent Technologies (Santa Clara, CA) 7890A gas chromatograph (GC) connected to a 5975C inert XL mass spectrometer (MS) for analysis using methods described before (Abraham et al., 2016). Statistical analyses were performed using GraphPad Prism software version 9.0.0 (GraphPad, San Diego, CA). Welch's one-way ANOVA was performed with an unpaired Welch's *t*-test per species, testing treatment group responses against solvent control responses. Outliers were removed by the robust regression and outlier removal (ROUT) method, where Q is the maximum false discovery rate set at 1%.

## Results

### The Presence of Specific LCOs and COs Alters *Laccaria bicolor* Growth, Hyphal Formation, and Clamp Connections

**Figure 1:**
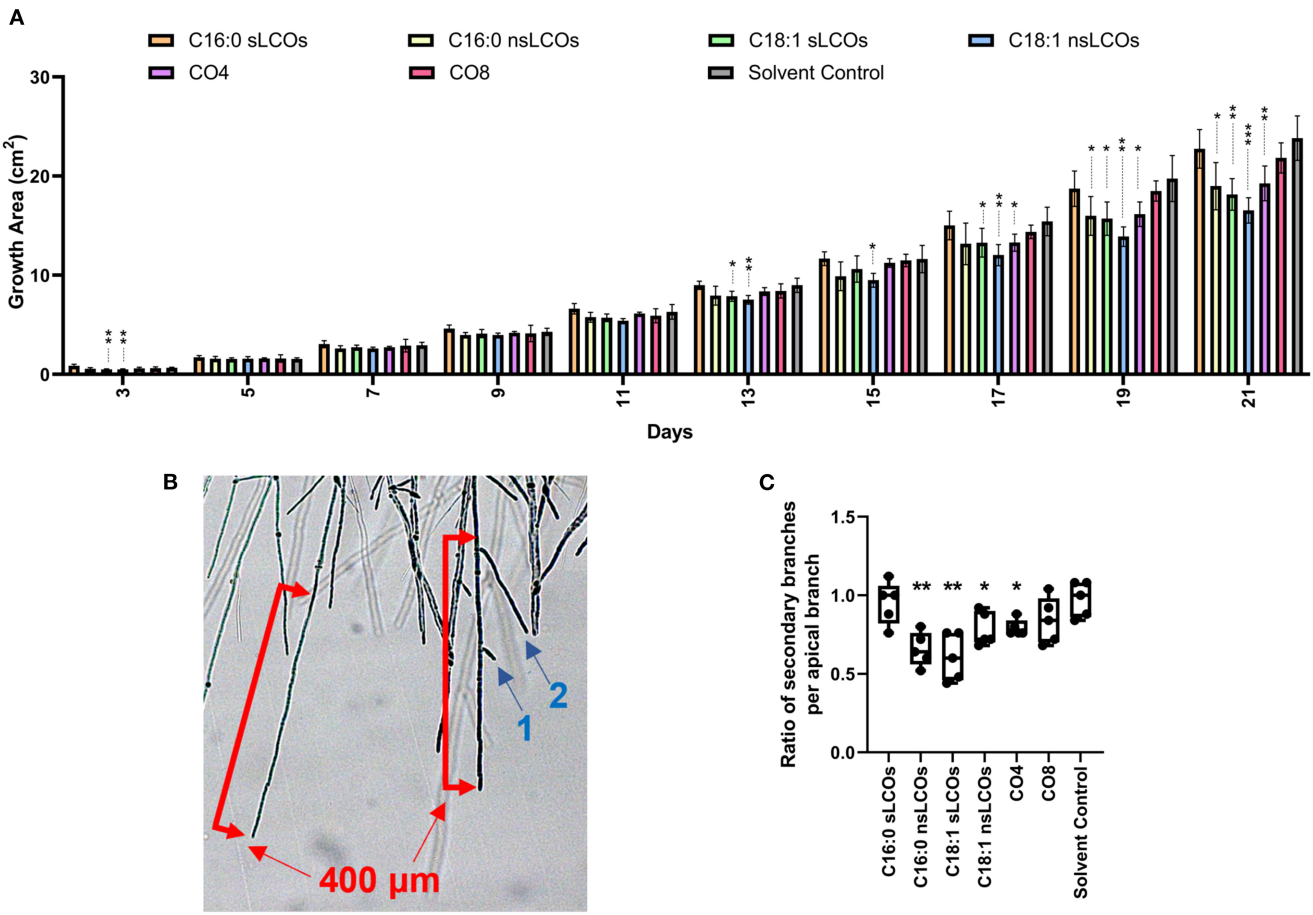
LCOs and COs effect on growth and hyphal branching. **(A)** Growth of *Laccaria bicolor* over 21 days when exposed to LCOs or COs. Diameter measurements were taken every other day, starting on the day 3. Fungal growth is the area without the 1 cm^2^ plug. Welch's ANOVA test was significant for 3 days post-inoculation (dpi) (*p* = 0.0058), 11 dpi (*p* = 0.0032), 13 dpi (*p* = 0.0079), 15 dpi (*p* = 0.01), 17 dpi (*p* = 0.0199), 19 dpi (*p* = 0.0005), and 21 dpi (*p* = 0.0004). Treatments were compared to the solvent controls with Welch's unpaired *t*-test. N is five biological replications. **(B)** Hyphal branching was determined by the average ratio of secondary branches (indicated by blue arrows and numbers) to 400 μm of the apical branch (red scale bar) counted after 3 dpi. **(C)** The ratios of secondary branches per apical branch in treatments were compared to the solvent controls with Welch's unpaired *t*-test (Welch's ANOVA test *p* = 0.0105). N is five biological replications with five technical replications each where five apical branches were counted. For all measurements containing significant treatments compared to the solvent control are as follows: (*) is a *p* < 0.05; (**) is a *p* < 0.01; (***) is a *p* < 0.001.

A panel of synthesized LCOs and COs were individually tested as additives to *L. bicolor* growth media, at a concentration of 10^−8^ M, to evaluate their impact on fungal growth. As observed in Figure 1A, the relative growth of treated samples compared to the solvent control was similar for ~11 days; however, from day 13 onwards, significant differences became apparent. The treated samples reduced radial growth compared to the solvent control. There was an exception for growth on day 3, where treatments, C18:1 sLCOs and nsLCOs, caused decreased radial growth compared to the solvent control. Overall, individually applied treated samples with CO4, C16:0 nsLCOs, C18:1 sLCOs and C18:1 nsLCOs compared to the solvent control had decreased radial growth.

Interestingly, the same treatments that resulted in reduced fungal growth also led to decreased branch density at 3 days post-inoculation (dpi) (Figures 1B,C). The only other known example of reduced branch density caused by these molecules is in *Aspergillus fumigatus* following exposure to exogenous C16:0 sLCOs between the concentrations of 10^−6^ M to 10^−12^ M, but not to other LCO or CO treatments as shown here in *L. bicolor* (Rush et al., 2020).

*Laccaria bicolor* strain S238N is dikaryotic, which is the predominant vegetative structure (Martin and Selosse, 2008). Moreover, this dikaryotic strain produces clamp connections at each septum to ensure the fungus is binucleate (Martin and Selosse, 2008). Clamp connections are hook-like structures and are unique to the phylum Basidiomycota (Alexopoulos et al., 1996). We investigated whether exogenous 10^−8^ M LCOs and COs influence clamp connection formation (Supplementary Figure S1A). At 7 dpi, C16:0 sLCOs treated samples had more clamp connections than the solvent control (Supplementary Figure S1B). At 14 dpi, C16:0 sLCOs, C16:0 nsLCOs, and C18:1 sLCOs treated samples had more clamp connections than the solvent control (Supplementary Figure S1C). However, this observation plateaued at 21 dpi (Supplementary Figure S1D).

### *Laccaria bicolor* Grown in the Presence of LCOs and COs Results in Distinct Proteomes

**Figure 2:**
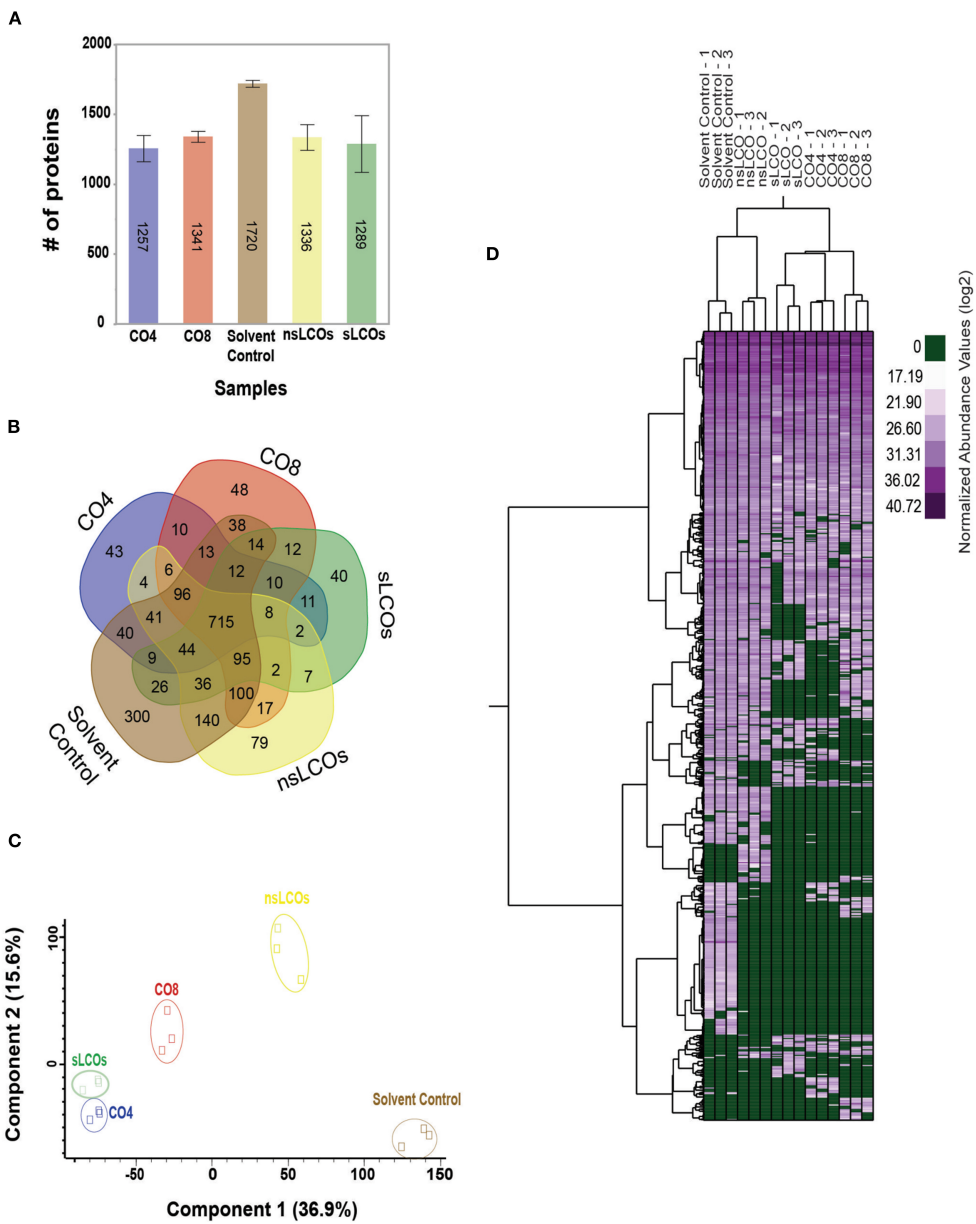
Proteomics analysis of *L. bicolor* under exposure of COs and LCOs. **(A)** Total number of proteins identified. **(B)** Venn diagram showing shared and unique proteins after data normalization and selection of proteins that were identified in at least 2 out of 3 biological replicates. **(C)** Principal component 2 with explained variance given as a percentage value after data normalization, filtration, and imputation. **(D)** Heatmap from the normalized abundance values of all identified proteins found in at least 2 out of 3 biological replicates per condition.

Given the observed phenotypic effects on *L. bicolor* growth and hyphal branching, bottom-up proteomic measurements were conducted for *L. bicolor* cells harvested 21 dpi with 10^−8^ M CO4, CO8, and mixtures of sLCOs or nsLCOs purified from rhizobia (see Materials and Methods). Overall, an average of 1,388 proteins was identified across each sample (Figure 2A), and a total of 715 proteins were identified in all samples (Figure 2B). A principal component analysis (PCA) revealed distinct groupings per sample (Figure 2C), with nsLCOs and solvent control sample clusters being closer to each other than with the remaining treatments. All quantifiable protein identifications are provided in Supplementary Table S1.

### LCOs and COs Lead to Highly Reduced *L. bicolor* Proteome Expression Profiles

Protein abundance changes between samples were evaluated by ANOVA followed by a Tukey's HSD test (Supplementary Table S1). Proteins with a *p* ≤ 0.05 and an absolute log2 fold-change difference ≥ 1 were characterized as significantly different between samples. A detailed list of regulated proteins is provided in Supplementary Table S2. The comparative proteomic analysis revealed that all treatments resulted in a substantial downregulation of proteome expression compared to solvent control samples (Figure 2D, Supplementary Figure S2). The overall reduced proteome expression profiles imply significant regulation in response to LCOs and COs treatments.

**Figure 3:**
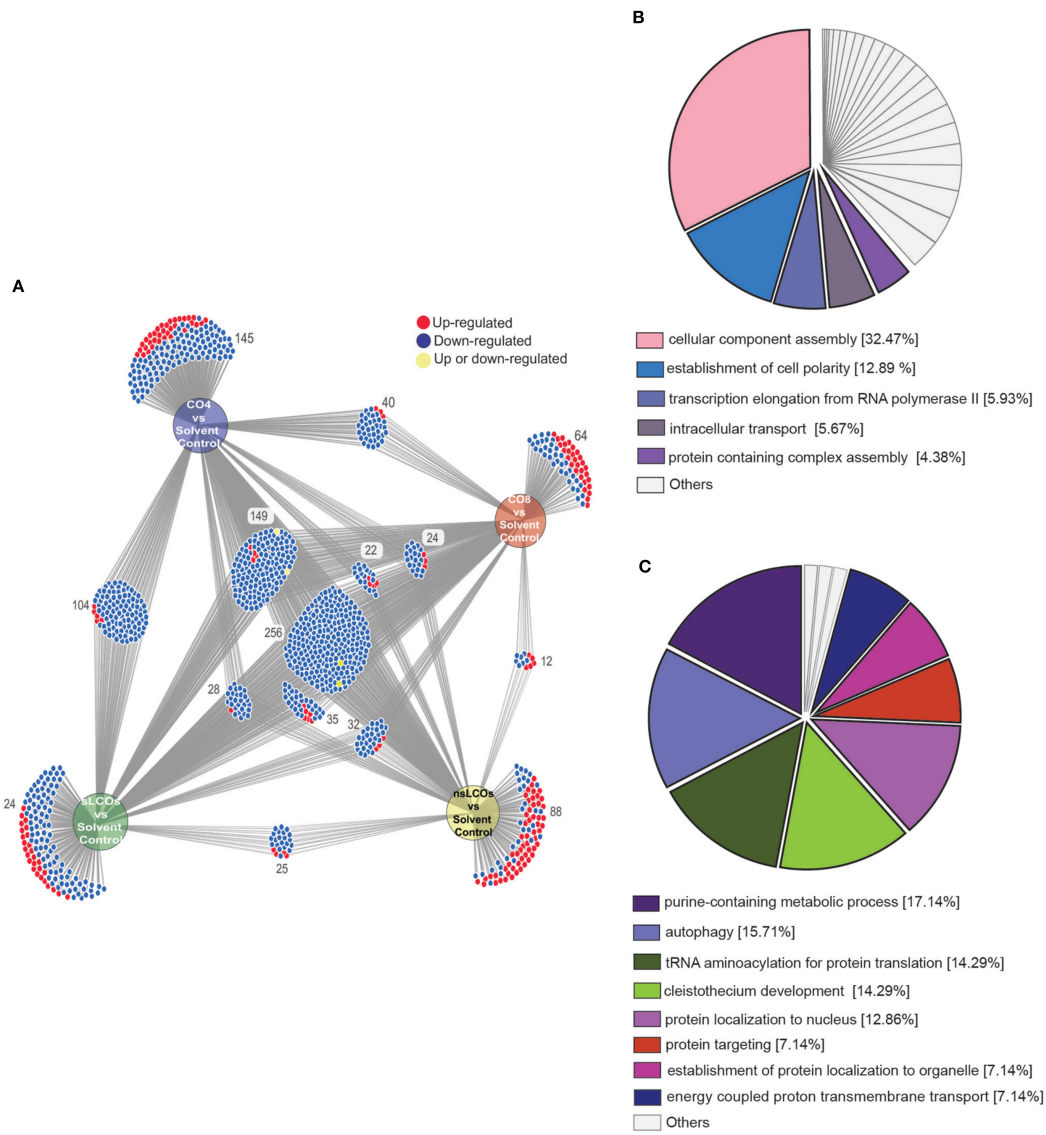
Proteome interrogation of *L. bicolor* growing with COs and LCOs compared to solvent control. **(A)** DiVenn diagram showing the numbers of up- (red color) and downregulated (blue color) proteins that are shared and/or unique in the comparisons between treatments and control samples. **(B)** GO enrichment analysis of the 254 common downregulated proteins shared between all treatments compared to control samples. **(C)** GO enrichment analysis of 144 common downregulated proteins in sLCO, CO4, and CO8-treated samples compared to control samples. Enrichment analyses performed with the Cytoscape plug-in ClueGO (Shannon et al., 2003; Bindea et al., 2009), terms reported were significant by Benjamini-Hochberg term *p* ≤ 0.05.

To further interrogate the differential protein expression data, the web-based tool DiVenn (Sun et al., 2019) was utilized to relate protein regulation levels between each pairwise comparison between treatments and solvent control. As shown in Figure 3A, protein regulation was quite similar across LCO and CO treatments with a relatively minor amount being unique to each treatment (average ~13%). 256 proteins were regulated across all treatments when compared to solvent control, and 254 of these were similarly downregulated. Additionally, CO4, CO8, and sLCOs overlapped with 144 similarly downregulated proteins when compared against solvent control, and these treatments also grouped closely in the provided PCA plot. Gene Ontology (GO) enrichment analyses were performed for both the 254 and 144 sets of downregulated proteins to identify significantly overrepresented GO terms (right-sided hypergeometric test, Benjamini-Hochberg corrected ps ≤ 0.05). The 244 proteins that were similarly regulated between all treatments included a substantial representation of protein function related to “cellular component assembly” and “establishment of cell polarity” (Figure 3B); whereas, the set of 144 proteins similarly regulated in CO4, CO8, and sLCOs treatments harbored functional categories such as “purine-containing metabolic process,” “autophagy,” “tRNA aminoacylation for protein translation,” and “cleistothecium development” (Figure 3C). In general, these overrepresented functions have associated implications with eukaryotic growth and development, response to stress, or adaptation to an environment (Klionsky, 2007; Raina and Ibba, 2014; Chua and Fraser, 2020; Aleklett and Boddy, 2021).

There was a little overlap between the different treatments when it comes to protein abundance in comparison to the solvent control. Nevertheless, the functional roles of upregulated protein abundances during treatment with LCOs and COs are associated with different aspects of fungal growth (Supplementary Table S3). Notable abundance changes during specific LCO and COs treatments occurred in processes such as actin organization and dynamics and cell wall remodeling. For example, several Ras proteins, thought to have a role in regulating cell wall synthesis in filamentous fungi (Kanauchi et al., 1997; Truesdell et al., 1999), were positively regulated in CO4-treated samples. Several glycoside hydrolases, which play essential roles in cell wall recycling and remodeling during specific conditions like carbon starvation (White et al., 2002; Van Munster et al., 2015), were upregulated in specific treatments. Fungal response to the presence of exogenous LCOs and COs also elicited the production of signaling molecules or defense mechanisms that *L. bicolor* employs to counteract/communicate with neighboring fungi or bacteria (Supplementary Table S3). Examples includ a terpenoid synthase (Jia et al., 2019) in sLCO treated samples, a Nudix hydrolase family (Dong and Wang, 2016) in CO4 treated samples, and Tectonin-2, a fungal protein with specificity for O-methylated glycans present in bacteria cell walls and nematode membranes (Wohlschlager et al., 2014; Sommer et al., 2018) identified in CO8 treated samples. Interestingly, an ectomycorrhiza-induced small, secreted protein (MISSP 11.8), implicated in plant-fungal interactions, was also among the upregulated proteins in CO8-treated samples. MiSSP proteins are known to be required to establish the symbiosis between plants and ECM forming fungi (Plett et al., 2014; Pellegrin et al., 2019; Daguerre et al., 2020; Kang et al., 2020). Currently, this is the first report of these small proteins being regulated in the absence of a host plant.

Not unexpectedly, many upregulated proteins did not have any assigned function (Supplementary Table S3). Sequence lengths of these proteins ranged from 101 to 1,327 amino acids. Further interrogation of these proteins with the SignalP-5.0 server (Armenteros et al., 2019) resulted in the prediction of signal peptides in just two of them, ID. B0CTV7 (245 aa) in sLCO and B0DKW4 (439 aa) in nsLCO-treated samples, thus suggesting that most of these proteins may be related to intracellular processes performed by *L. bicolor*. Nevertheless, the expression of these proteins in the presence of COs or LCOs is an avenue that could be considered in future studies looking to expand the functional annotation repertoire of the *L. bicolor* genome.

### Decreased Amounts of Organic and Fatty Acids Were Identified in CO- and LCO-Treated Samples

Gas chromatography-mass spectrometry measurements were collected for *L. bicolor* cells harvested at 21 dpi with specific COs or LCOs treatments. These analyses yielded a total of 62 quantifiable metabolites, from which 10 were significantly differentially regulated compared to the solvent control samples (Welch's ANOVA *p* < 0.05) (Supplementary Figure S3). An exception was oxalic acid which had a *p* = 0.0502, and stearic acid which had a *p* = 0.0533. There would be no additional significant metabolites to report if the *p* was adjusted to <0.07. Despite these metabolites not having a *p* < 0.05, they have biological meaning in the fungus lifestyle. There were differential regulations of four organic acids (citric, malic, oxalic, and lactic), which play a role in giving filamentous fungi a competitive advantage over other microbes (Liaud et al., 2014). Citric, malic, and oxalic acids were downregulated whereas lactic acid was upregulated in treated samples. Three fatty acids (palmitic, stearic, and tetracosanoic), that play important roles as signaling molecules in fungi, were all downregulated compared to solvent control samples. Both sLCOs and nsLCOs had a significant downregulation of trehalose 6-phosphate, a precursor of the known fungal storage carbohydrate (trehalose), and glucose 6-phosphate, a known storage for glycogen (Tschaplinski et al., 2014). Additionally, two amine compounds and an unidentified compound at retention time 10.07 min and mass-to-charge (*m/z*) fragments of 172, 271, and 256 *m/z* were also significantly downregulated in almost all treatments.

## Discussion

**Figure 4:**
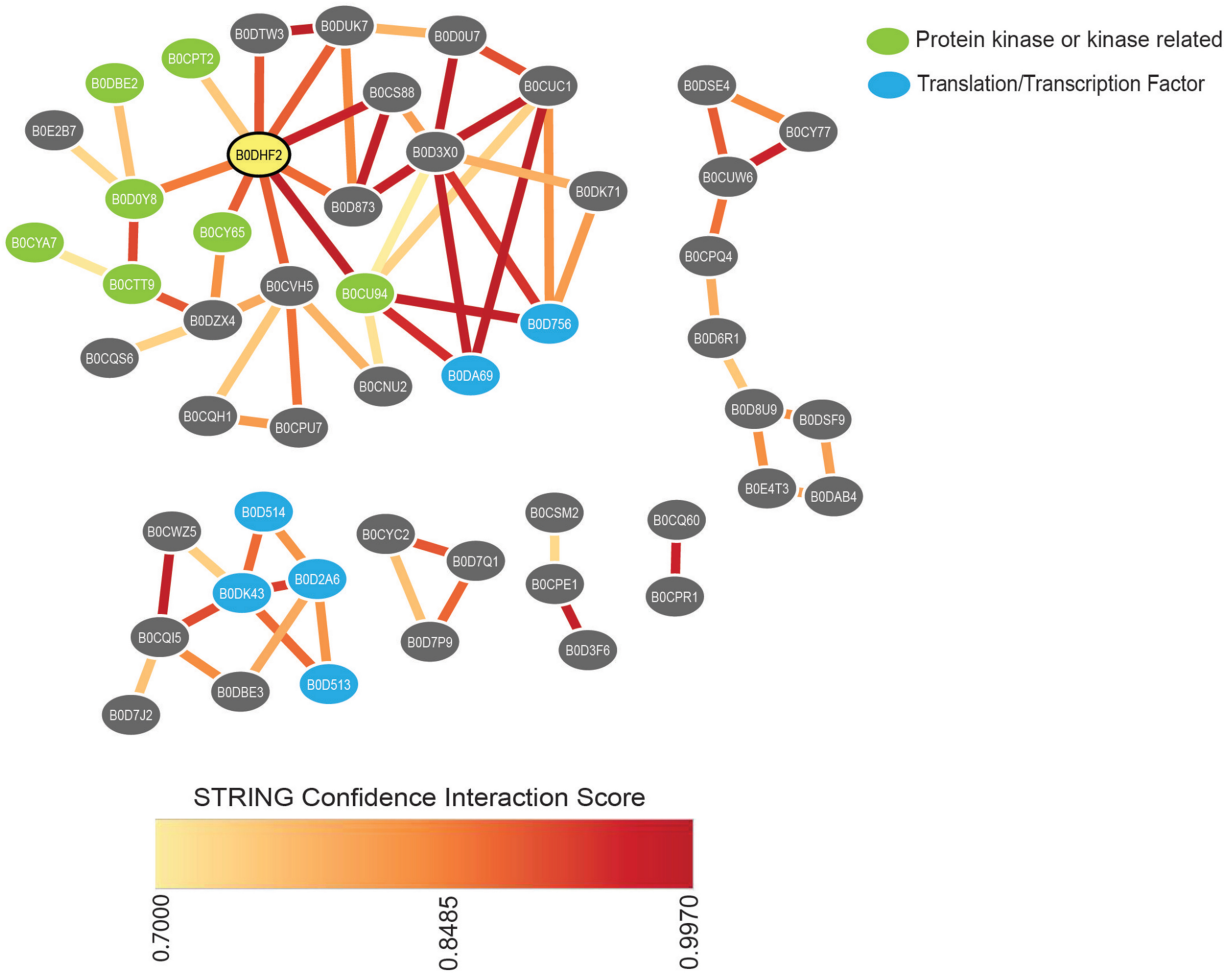
STRING network of all common downregulated proteins in treatments compared to control samples belonging to “cellular component assembly” and “establishment of cell polarity” GO categories. The network summarizes the predicted associations between proteins found by the STRING database. Each protein is represented by a node and the edges represent predicted functional associations. Edge colors indicate the confidence interaction score based on different types of evidence considered by STRING. Only protein-protein associations with high confidence scores (≥ 0.7) were considered in the predicted network. The node representing the CMGC/CDK/CDC2 protein kinase is highlighted in yellow. Disconnected proteins in the network are not shown.

The integrated characterization of *L. bicolor* physiology with molecular and metabolic measurements provides new insights into how fungal behavior is altered in the presence of LCOs and COs. Moreover, the in-depth proteome analysis serves as a crucial data resource and a first step toward understanding how these secondary metabolites influence fungal behavior in the absence of a host. Overall, each treatment decreased the total number of proteins, with nsLCOs being the treatment that had the least impact 21 days post-induction (dpi). The underlying protein regulation and associated functional annotations might explain the reduced fungal growth compared to solvent control samples. Exposure of *L. bicolor* to LCOs or COs treatments resulted in the downregulation of proteins involved in polarized growth compared to solvent control samples. These proteins were part of “cellular component assembly” and “establishment of cell polarity” GO categories that included kinases, transcription factors, GTPases, nuclear transport-related proteins, dynein chains, and others (Supplementary Table S2). Network-based integration of these proteins using protein-protein interactions in the STRING v11.5 database suggested biologically meaningful clusters of proteins, as observed in Figure 4. Amongst these, one cluster showed the highest number of protein-protein connections and contained several protein kinases. We noticed the presence of a protein annotated as a CMGC/CDK/CDC2 protein kinase (Accession ID. B0DHF2), which was the most connected node in the resulting network (Supplementary Table S4). In general, cyclin-dependent kinases (Cdks) are essential for hyphal development under specific hypha-inducing conditions and transcription of hypha-specific genes (Loeb et al., 1999; Chen et al., 2000). In *C. albicans*, disruption of a gene coding for a CDC2-related kinase (Crk1) have led to defective hyphal formation under various hypha-inducing conditions suggesting an active role in the transcriptional regulation of hypha-specific genes (Chen et al., 2000). In *S. cerevisiae*, the activity of another Cdk, CDC28, has been shown to regulate various aspects of filamentous growth depending on its associated cyclins (Edgington et al., 1999; Loeb et al., 1999; Rua et al., 2001). A closer look in sequence space of the *L. bicolor* CMGC/CDK/CDC2 protein kinase identified here revealed a higher level of similarity to CDC28 from *S. cerevisiae* (67% similarity) than to the reported Crk1 in *C. albicans* (23% similarity) and the visualization provided by the STRING network seems to suggest that this protein is critical to initiate a cascade of phosphorylation events that might lead to the regulation of polarized growth. Interestingly, although not connected to the kinases mentioned previously, another cluster of proteins contained several translation-initiation factors, strengthening the idea of this regulatory network (Figure 4).

Additionally, all treated samples caused several small GTPases to be downregulated compared to solvent control samples (Supplementary Table S2). These proteins play essential roles as molecular switches that can turn various cell polarity pathways on or off (Ranocchi and Amicucci, 2021). In *S. cerevisiae*, evidence has suggested that CDK complexes act as crucial regulators of small GTPases. For example, for the Rho family GTPase Cdc42, which is considered a master player in the process of yeast budding and its associated polarized growth (Johnson, 1999; Park and Bi, 2007), it is hypothesized that CDKs exert control on it by either direct phosphorylation or by regulation of its effectors and adaptor proteins (Pedraza et al., 2019). Although our STRING analysis did not show any evidence of interaction between protein kinases and small GTPases, the identifications provided in this study should be considered for investigations into potential associations between these groups of enzymes in *L. bicolor*. Additionally, other proteins related to filamentous growth development were downregulated in all-treated samples. Amongst these, we identified dynein heavy chains. Dynein is a type of cytoskeleton-dependent motor protein that has various roles in transporting proteins, vesicles, and organelles (Yamamoto and Hiraoka, 2003; Xiang and Fischer, 2004; Fischer et al., 2008). In filamentous fungi, the disruption of dynein expression causes defects in typical fungal developmental structures (Seiler et al., 1999; Riquelme et al., 2002).

The determination of the specific regulatory network underlying the effects on *L. bicolor* polarized growth when exposed to LCOs or COs is out of the scope of this manuscript; however, these observations suggest that the growth effects observed here may be orchestrated by the coordinated actions of protein kinases, small GTPases and other effectors. To our knowledge, nutrient starvation is not playing a significant role in these molecular responses, as all samples kept growing past 21 days. The induction of hyphal branching in response to plant or fungal signals has been documented. However, the only other study showing that exogenous LCOs signals can actively suppress filamentous branching in fungi was the study mentioned previously, investigating the effects of LCOs or COs in non-symbiotic fungi (Rush et al., 2020). It is plausible that the production of exogenous LCOs or COs by other microbes in the vicinity of *L. bicolor* acts to counteract the spatial growth of the fungus in some sort of competition for resources. This hypothesis is also supported by the upregulation of *L. bicolor* proteins involved in cross-communication or defense response mechanisms observed in specific treatments, like a terpenoid synthase in sLCO-treated samples, tectonin-2 in CO8-treated samples, or an isochorismatase family protein in nsLCO-treated samples. The latter is part of a group of enzymes speculated to reduce external responses such as the accumulation of salicylic acid secreted by plants in response to pathogen attack (Soanes et al., 2008).

The treatments that resulted in the most significant growth effects (CO4, CO8, and sLCOs), shared protein regulation in processes related to the repression of polarized growth in *L. bicolor*, such as proteins associated with the GO categories “autophagy” and “cleistothecium development” (Supplementary Table S2). In general, autophagy is an essential intracellular turnover mechanism involving the lysosomal/vacuolar pathway (Levine and Klionsky, 2004; Pollack et al., 2009). In filamentous fungi, autophagy appears to be involved in nutrient recycling during starvation and normal developmental processes (Pollack et al., 2009). In several fungal species, autophagy has been implicated in determining cell architecture during differentiation and development (Levine and Klionsky, 2004) and morphogenesis and morphology (Pollack et al., 2009). Few examples of downregulated proteins associated with the autophagy category include a WD40 repeat like-protein (Uniprot ID. B0DVN6) with 35% sequence identity to the *S. cerevisiae* autophagy-related gene protein ATG18, alongside vacuolar protein sorting-associated proteins (VPS) VPS13 (B0CPT6), VPS1 (B0D3D9), and a GTP-binding protein ypt (B0CQK4) with sequence identities above 30% to VPS13, VPS1, and VPS21 in yeast, respectively. ATGs have been intensively investigated in *S. cerevisiae*, and up to 42 proteins are known. Among these, 18 ATG have been previously implicated in different steps of autophagy (Lin et al., 2019). For example, yeast ATG18 is involved in vesicle nucleation, whereby proteins and lipids concentrate on forming the pre-autophagosomal structure. VPS proteins act as controllers directing the specific delivery of cellular material targeted for vacuole degradation (Kim et al., 2015). In filamentous fungi, such as *Fusarium graminearum* and *Magnaporthe oryzae*, mutants lacking VPS74 were strikingly reduced in hyphal growth and fungal virulence on host plants. Both mutants showed abnormal hyphal morphology with swollen hyphal tips (Kim et al., 2015). As such, the observed downregulation of VPS proteins implies that the exogenous application of specific COs and LCOs could alter cellular trafficking and sorting processes that are important for the delivery of matrix components necessary for hyphal elongation (Wessels, 1990).

Among regulated proteins associated with the “cleistothecium development” GO term there were several 26S proteasome subunits. “Cleistothecium development” category may be an artifact of the gene annotation, since *L. bicolor* is a basidiomycete and will not develop a cleistothecium. A previous study in yeast filamentous fungi suggested that the 26S proteasome controls filamentous-form cell properties through the regulation of the Rpn4 subunit protein. When the RPN4 gene was deleted in this organism, cells grow slower compared to the wild type (Prinz et al., 2004). Although we did not detect in our study the Rpn4 protein specifically, we observed downregulation of other 26S proteasome subunits, including P43, Rpn1, and Rpn7. The latter was also reported to be significantly induced in filamentous forms of yeast cells (Prinz et al., 2004). Thus, it is plausible to support the idea that in *L. bicolor*, similarly to yeast, the induction of specific proteasomal subunits could exert different types of regulatory control on filamentous growth.

In addition to the reported observations at the proteome level, metabolite analysis provided another layer of evidence supporting the suppression of *L. bicolor* filamentous growth. In this study, the abundance of three fatty acids (palmitic, stearic, and tetracosanoic acids) was significantly lower in some LCOs and COs treatments compared to the solvent control samples (Supplementary Figure S3). Fatty acids are components of membranes and storage lipids. In filamentous fungi, many lipids accumulate in hyphae as they tend to be used as carbon and energy sources during starvation (Laczko et al., 2004; Reich et al., 2009). Thus, it is plausible to hypothesize that the downregulation of fatty acids observed here may be contributing to an alteration in the production of material necessary for the formation of new hyphae (i.e., new membrane). Interestingly, these metabolite observations can be linked to the downregulation of the fatty acid synthase (FAS) and acetyl-CoA carboxylase (ACC), two enzymes required for the stepwise synthesis of fatty acids in *L. bicolor* (Reich et al., 2009).

Low molecular weight organic acids (LMWOAs) are commonly found in rhizospheric exudates and play a role in soil fertility, plant growth, and organization of microbial communities (Macias-Benitez et al., 2020). For mycorrhizal forming fungi, the expression of LMWOAs are dependent on different environmental conditions (Cumming et al., 2001; Eldhuset et al., 2007; Machuca et al., 2007; Johansson et al., 2008). For example, it has been proposed that the production of organic acids like citric, malate, citrate, and oxalate by mycorrhizal forming fungi can play essential roles in metal complexation, which helps in improved metal resistance (Plassard and Fransson, 2009). In our study, differential production of some organic acids was observed. The production of citric, malic, and oxalic acid was downregulated in almost all treatments compared to solvent control samples. In contrast, lactic acid was upregulated in both LCO treatments (Supplementary Figure S3). Alternative to the metal complexation hypothesis, the regulation of organic acids might be recruiting beneficial microbes as previously shown (Rodriguez-Morgado et al., 2017; Macias-Benitez et al., 2020), yet this potential association garners further investigation.

As more investigations are conducted on COs and LCOs, the roles of these molecules in fungal biology have expanded. They were first characterized as the primary signals allowing root colonization by rhizobia or mycorrhizal fungi. Now LCOs were shown to have a great influence on fungal development even in organisms with no known ability to form symbiotic associations with plants (Rush et al., 2020; Khokhani et al., 2021). The observations reported here in *L. bicolor* suggest that a cascade of phosphorylation events are potentially responsible for the reduced growth observed in samples grown 21 dpi in presence of LCOs and COs.

In the *L. bicolor*—*Populus* sp. ectomycorrhizal association, the application of sLCOs but not nsLCOs was reported to increase the ratio of mantle width to root diameter as well the ratio of Hartig net boundary to root circumference (Cope et al., 2019). This observation was puzzling as *Populus* sp. is known to be more sensitive to nsLCOs than sLCOs (Cope et al., 2019, 2021). Given the data presented in this manuscript, it is likely that this result was potentially due to the effect of sLCOs on *L. bicolor* rather than on its host plant.

**Figure 5:**
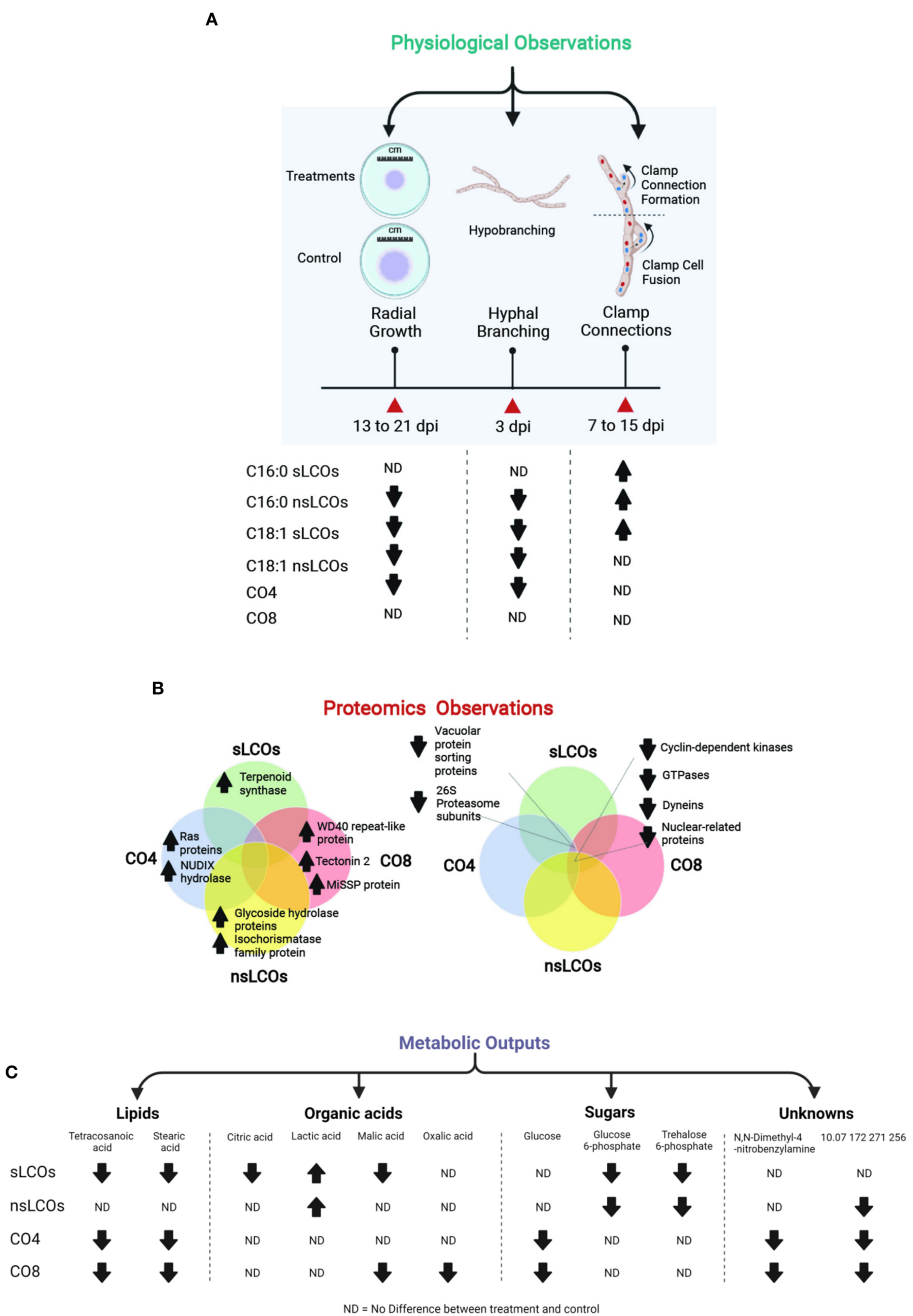
Summary of the main physiological, proteomic, and metabolic changes observed in *L. bicolor* when exposed to COs and LCOs compared to control samples. **(A)** Reduced hyphal growth was observed in samples grown with nsLCOs, C18:1 sLCOs and CO4. **(B)** Most upregulated proteins quantified in this study were unique to each treatment and included cases of molecules involved in fungal growth control like Ras proteins, as well as proteins involved in mechanisms of cross-communication between fungi and other organisms; instead, the vast majority of downregulated proteins were found to be shared between treatments such as cyclin-dependent kinases, GTPases, and dyneins. Other examples discussed through this manuscript are shown. **(C)** Ten metabolites were significantly regulated in treated *L. bicolor* samples compared to controls and included cases of organic acid, fatty acids, sugars, and metabolites with unknown functions.

Here we suggest that specific LCOs and COs are possibly fungistatic agents that inhibit the growth of the fungus without killing it (Figure 5). Therefore, it can be the reason why most fungi produce LCOs (Rush et al., 2020) to inhibit the growth of nearby microbes and explain why the growth and development of some fungi are influenced by certain LCOs and not others (Marburger et al., 2018; Rush et al., 2020). Surprisingly, we observed that the LCOs predominantly produced by the same organism have the most significant influence on it (Rush et al., 2020). That was the C16:0 LCOs in *A. fumigatus* (Rush et al., 2020) and the C18:1 LCOs as reported here in *L. bicolo*r. These observations remain inexplicable as why a fungus would produce a fungistatic compound to inhibit its growth. Additional data are needed to support or disprove this suggestion. Lastly, further experiments are required to determine if these changes in fungal development are not xenobiotic responses since sLCOs and nsLCOs were purified from rhizobia (Maillet et al., 2011; Mukherjee and Ane, 2011; Sun et al., 2015; Cope et al., 2019).

The results presented here provide baseline evidence that an LCO-producing organism might be controlled by its own chemical signal. To understand these mechanisms better, it would be ideal to generate mutant strains in which the genes responsible for LCO production are overexpressed or knocked out. This would allow to examine the behavior of the fungus in the absence or overproduction of its own LCOs. However, the genes responsible for LCO production in fungi have not been characterized yet. To conclude, this study document potential role for LCOs outside of symbiosis and open new avenues for research on the biological function of LCOs and their implication in fungal growth and development.

## Author's Note

This manuscript has been authored by Q21 UT-Battelle, LLC under Contract No. DE-AC05- 00OR22725 with the U.S. Department of Energy. The Department of Energy will provide public access to these results of federally sponsored research in accordance with the DOE Public Access Plan (http://energy.gov/downloads/doe-public-access-plan).

## Data Availability Statement

All proteomics MS/MS raw and searched data files used in this study were deposited at the 574 MassIVE repository (https://massive.ucsd.edu/) as public dataset MSV000088201.

## Author Contributions

TR, PA, RH, and JL planned and designed the research. SC, SF, JM, and JMA provided LCO molecules. TR and MS did the inoculations and provided samples for proteomics and metabolomics analysis. TR performed all *L. bicolor* growth experiments and observations. MV performed all proteomics sample preparation and analysis and wrote the manuscript. NE and TT performed metabolite extraction and analysis. All authors contributed to the revision of the manuscript.

## Funding

This research was sponsored by the Genomic Science Program, US Department of Energy (DOE), Office of Science, Biological and Environmental Research, as part of the Plant Microbe Interfaces Scientific Focus Areas at the Oak Ridge National Laboratory (ORNL). ORNL is managed by UT-Battelle LLC for DOE under contract DE-AC05-00OR22725. This work was also supported by the NSF award # 1546742 as well as USDA Hatch #WIS03041 to JMA.

## Conflict of Interest

This study received funding from the Genomic Science Program, US Department of Energy (DOE), Office of Science, Biological and Environmental Research, as part of the Plant Microbe Interfaces Scientific Focus Areas at the Oak Ridge National Laboratory (ORNL). ORNL is managed by UT-Battelle LLC for DOE under contract DE-AC05-00OR22725. This work was also supported by the NSF award # 1546742 as well as USDA Hatch #WIS03041 to JMA. Lastly, partial financial support from the LABEX ARCANE and CBH-EUR-GS (ANR-17-561 EURE-0003), Glyco@Alps (ANR-15-IDEX-02), and PolyNat Carnot Institut (ANR-16-CARN562 0025-01) for SF and SC. The funder was not involved in the study design, collection, analysis, interpretation of data, the writing of this article, or the decision to submit it for publication. The remaining authors declare that the research was conducted in the absence of any commercial or financial relationships that could be construed as a potential conflict of interest.

## Publisher's Note

All claims expressed in this article are solely those of the authors and do not necessarily represent those of their affiliated organizations, or those of the publisher, the editors and the reviewers. Any product that may be evaluated in this article, or claim that may be made by its manufacturer, is not guaranteed or endorsed by the publisher.

## References

[B1] Abraham P. E. Yin H. Borland A. M. Weighill D. Lim S. D. De Paoli H. C.. (2016). Transcript, protein and metabolite temporal dynamics in the CAM plant *Agave*. Nat Plants 2:16178. 10.1038/nplants.2016.178 27869799

[B2] Aleklett K. Boddy L. (2021). Fungal behaviour: a new frontier in behavioural ecology. Trends Ecol. Evol. 36, 787–796. 10.1016/j.tree.2021.05.006 34172318

[B3] Alexopoulos C. J. Mims C. W. Blackwell M. (1996). Introductory Mycology. Hoboken, NJ: John Wiley and Sons.

[B4] Armenteros J. J. A. Tsirigos K. D. Sonderby C. K. Petersen T. N. Winther O. Brunak S.. (2019). SignalP 5.0 improves signal peptide predictions using deep neural networks. Nat. Biotechnol. 37, 420–423. 10.1038/s41587-019-0036-z 30778233

[B5] Bindea G. Mlecnik B. Hackl H. Charoentong P. Tosolini M. Kirilovsky A.. (2009). ClueGO: a Cytoscape plug-in to decipher functionally grouped gene ontology and pathway annotation networks. Bioinformatics 25, 1091–1093. 10.1093/bioinformatics/btp101 19237447 PMC2666812

[B6] Bonfante P. Anca I. A. (2009). Plants, mycorrhizal fungi, and bacteria: a network of interactions. Annu. Rev. Microbiol. 63, 363–383. 10.1146/annurev.micro.091208.073504 19514845

[B7] Bonfante P. Genre A. (2010). Mechanisms underlying beneficial plant-fungus interactions in mycorrhizal symbiosis. Nat. Commun. 1:48. 10.1038/ncomms1046 20975705

[B8] Buendia L. Girardin A. Wang T. M. Cottret L. Lefebvre B. (2018). LysM receptor-like kinase and LysM receptor-like protein families: an update on phylogeny and functional characterization. Front. Plant Sci. 9:1531. 10.3389/fpls.2018.01531 30405668 PMC6207691

[B9] Buendia L. Wang T. Girardin A. Lefebvre B. (2016). The LysM receptor-like kinase Sl LYK 10 regulates the arbuscular mycorrhizal symbiosis in tomato. New Phytol. 210, 184–195. 10.1111/nph.13753 26612325

[B10] Chabaud M. Genre A. Sieberer B. J. Faccio A. Fournier J. Novero M.. (2011). Arbuscular mycorrhizal hyphopodia and germinated spore exudates trigger Ca2+ spiking in the legume and nonlegume root epidermis. New Phytol. 189, 347–355. 10.1111/j.1469-8137.2010.03464.x 20880223

[B11] Chen J. Y. Zhou S. Wang Q. Chen X. Pan T. Liu H. P. (2000). Crk1, a novel Cdc2-related protein kinase, is required for hyphal development and virulence in *Candida albicans*. Mol. Cell. Biol. 20, 8696–8708. 10.1128/MCB.20.23.8696-8708.2000 11073971 PMC86484

[B12] Choi J. Summers W. Paszkowski U. (2018). Mechanisms underlying establishment of arbuscular mycorrhizal symbioses. Annu. Rev. Phytopathol. 56 56, 135–160. 10.1146/annurev-phyto-080516-035521 29856935

[B13] Chua S. M. H. Fraser J. A. (2020). Surveying purine biosynthesis across the domains of life unveils promising drug targets in pathogens. Immunol. Cell Biol. 98, 819–831. 10.1111/imcb.12389 32748425

[B14] Cope K. R. Bascaules A. Irving T. B. Venkateshwaran M. Maeda J. Garcia K.. (2019). The ectomycorrhizal fungus *Laccaria bicolor* produces lipochitooligosaccharides and uses the common symbiosis pathway to colonize *populus* roots. Plant Cell 31, 2386–2410. 10.1105/tpc.18.00676 31416823 PMC6790088

[B15] Cope K. R. Irving T. B. Chakraborty S. Ane J. M. (2021). Perception of lipo-chitooligosaccharides by the bioenergy crop *Populus*. Plant Signal. Behav. 16:1903758. 10.1080/15592324.2021.1903758 33794743 PMC8143229

[B16] Cumming J. R. Swiger T. D. Kurnik B. S. Panaccione D. G. (2001). Organic acid exudation by *Laccaria bicolor* and *Pisolithus tinctorius* exposed to aluminum *in vitro*. Canad. J. Forest Res. 31, 703–710. 10.1139/x00-203

[B17] Daguerre Y. Basso V. Hartmann-Wittulski S. Schellenberger R. Meyer L. Bailly J.. (2020). The mutualism effector MiSSP7 of *Laccaria bicolor* alters the interactions between the poplar JAZ6 protein and its associated proteins. Sci. Rep. 10:20362. 10.1038/s41598-020-76832-6 33230111 PMC7683724

[B18] Dénarié J. Debelle F. Prome J. C. (1996). Rhizobium lipo-chitooligosaccharide nodulation factors: signaling molecules mediating recognition and morphogenesis. Annu. Rev. Biochem. 65, 503–535. 10.1146/annurev.bi.65.070196.002443 8811188

[B19] D'Haeze W. Holsters M. (2002). Nod factor structures, responses, and perception during initiation of nodule development. Glycobiology 12, 76R−105R. 10.1093/glycob/12.6.79R 12107077

[B20] Dong S. Wang Y. (2016). Nudix effectors: a common weapon in the arsenal of plant pathogens. PLoS Pathog. 12:e1005704. 10.1371/journal.ppat.1005704 27737001 PMC5063578

[B21] Edgington N. P. Blacketer M. J. Bierwagen T. A. Myers A. M. (1999). Control of *Saccharomyces cerevisiae* filamentous growth by cyclin-dependent kinase Cdc28. Mol. Cell. Biol. 19, 1369–1380. 10.1128/MCB.19.2.1369 9891070 PMC116065

[B22] Eldhuset T. D. Swensen B. Wickstrom T. Wollebaek G. (2007). Organic acids in root exudates from *Picea abies* seedlings influenced by mycorrhiza and aluminum. J. Plant Nutr. Soil Sci. 170, 645–648. 10.1002/jpln.20070000525855820

[B23] Feng F. Sun J. Radhakrishnan G. V. Lee T. Bozsóki Z. Fort S.. (2019). A combination of chitooligosaccharides and lipochitooligosaccharides recognition promotes arbuscular mycorrhizal associations in *Medicago truncatula*. Nat. Commun. 10:5047. 10.1038/s41467-019-12999-5 31695035 PMC6834629

[B24] Fischer R. Zekert N. Takeshita N. (2008). Polarized growth in fungi - interplay between the cytoskeleton, positional markers and membrane domains. Mol. Microbiol. 68, 813–826. 10.1111/j.1365-2958.2008.06193.x 18399939

[B25] Genre A. Chabaud M. Balzergue C. Puech-Pagès V. Novero M. Rey T.. (2013). Short-chain chitin oligomers from arbuscular mycorrhizal fungi trigger nuclear C a2+ spiking in *Medicago truncatula* roots and their production is enhanced by strigolactone. New Phytol. 198, 190–202. 10.1111/nph.12146 23384011

[B26] Jeffries P. Gianinazzi S. Perotto S. Turnau K. Barea J. M. (2003). The contribution of arbuscular mycorrhizal fungi in sustainable maintenance of plant health and soil fertility. Biol. Fertil. Soils 37, 1–16. 10.1007/s00374-002-0546-5

[B27] Jia Q. D. Chen X. L. Kollner T. G. Rinkel J. Fu J. Y. Labbe J.. (2019). Terpene synthase genes originated from bacteria through horizontal gene transfer contribute to terpenoid diversity in fungi. Sci. Rep. 9:9223. 10.1038/s41598-019-45532-1 31239482 PMC6592883

[B28] Johansson E. M. Fransson P. M. A. Finlay R. D. Van Hees P. A. W. (2008). Quantitative analysis of exudates from soil-living basidiomycetes in pure culture as a response to lead, cadmium and arsenic stress. Soil Biol. Biochem. 40, 2225–2236. 10.1016/j.soilbio.2008.04.016

[B29] Johnson C. W. Abraham P. E. Linger J. G. Khanna P. Hettich R. L. Beckham G. T. (2017). Eliminating a global regulator of carbon catabolite repression enhances the conversion of aromatic lignin monomers to muconate in *Pseudomonas putida* KT2440. Metab. Eng. Commun. 5, 19–25. 10.1016/j.meteno.2017.05.002 29188181 PMC5699531

[B30] Johnson D. I. (1999). Cdc42: an essential Rho-type GTPase controlling eukaryotic cell polarity. Microbiol. Mol. Biol. Rev. 63, 54–105. 10.1128/MMBR.63.1.54-105.1999 10066831 PMC98957

[B31] Kall L. Canterbury J. D. Weston J. Noble W. S. Maccoss M. J. (2007). Semi-supervised learning for peptide identification from shotgun proteomics datasets. Nat. Methods 4, 923–925. 10.1038/nmeth1113 17952086

[B32] Kamel L. Keller-Pearson M. Roux C. Ane J. M. (2017). Biology and evolution of arbuscular mycorrhizal symbiosis in the light of genomics. New Phytol. 213, 531–536. 10.1111/nph.14263 27780291

[B33] Kanauchi A. Yamashiro C. T. Tanabe S. Murayama T. (1997). A ras homologue of *Neurospora crassa* regulates morphology. Mol. General Genet. 254, 427–432. 10.1007/s004380050435 9180696

[B34] Kang H. Chen X. Kemppainen M. Pardo A. G. Veneault-Fourrey C. Kohler A.. (2020). The small secreted effector protein MiSSP7.6 of *Laccaria bicolor* is required for the establishment of ectomycorrhizal symbiosis. Environ. Microbiol. 22, 1435–1446. 10.1111/1462-2920.14959 32090429

[B35] Khokhani D. Carrera Carriel C. Vayla S. Irving T. B. Stonoha-Arther C. Keller N. P.. (2021). Deciphering the Chitin Code In Plant Symbiosis, Defense, And Microbial Networks. Annu. Rev. Microbiol. 75, 583–607. 10.1146/annurev-micro-051921-114809 34623896

[B36] Kidaj D. Wielbo J. Skorupska A. (2012). Nod factors stimulate seed germination and promote growth and nodulation of pea and vetch under competitive conditions. Microbiol. Res. 167, 144–150. 10.1016/j.micres.2011.06.001 21723717

[B37] Kim H. K. Kim K. W. Yun S. H. (2015). Multiple roles of a putative vacuolar protein sorting associated protein 74, FgVPS74, in the cereal pathogen *Fusarium graminearum*. J. Microbiol. 53, 243–249. 10.1007/s12275-015-5067-7 25845538

[B38] Klionsky D. J. (2007). Autophagy: from phenomenology to molecular understanding in less than a decade. Nat. Rev. Mol. Cell Biol. 8, 931–937. 10.1038/nrm2245 17712358

[B39] Kuchitsu K. Yazaki Y. Sakano K. Shibuya N. (1997). Transient cytoplasmic pH change and ion fluxes through the plasma membrane in suspension-cultured rice cells triggered by N-acetylchitooligosaccharide elicitor. Plant Cell Physiol. 38, 1012–1018. 10.1093/oxfordjournals.pcp.a029265

[B40] Labbe J. L. Weston D. J. Dunkirk N. Pelletier D. A. Tuskan G. A. (2014). Newly identified helper bacteria stimulate ectomycorrhizal formation in *Populus*. Front. Plant Sci. 5:579. 10.3389/fpls.2014.00579 25386184 PMC4208408

[B41] Laczko E. Boller T. Wiemken V. (2004). Lipids in roots of *Pinus sylvestris* seedlings and in mycelia of *Pisolithus tinctorius* during ectomycorrhiza formation: changes in fatty acid and sterol composition. Plant Cell Environ. 27, 27–40. 10.1046/j.0016-8025.2003.01122.x

[B42] Lerouge P. Roche P. Faucher C. Maillet F. Truchet G. Prom,é J. C.. (1990). Symbiotic host-specificity of *Rhizobium meliloti* is determined by a sulphated and acylated glucosamine oligosaccharide signal. Nature 344, 781–784. 10.1038/344781a0 2330031

[B43] Levine B. Klionsky D. J. (2004). Development by self-digestion: molecular mechanisms and biological functions of autophagy. Dev. Cell 6, 463–477. 10.1016/S1534-5807(04)00099-1 15068787

[B44] Liaud N. Giniés C. Navarro D. Fabre N. Crapart S. Gimbert I. H.-. (2014). Exploring fungal biodiversity: organic acid production by 66 strains of filamentous fungi. Fungal Biol. Biotechnol. 1, 1–10. 10.1186/s40694-014-0001-z26457194

[B45] Lin H. Y. Wang J. J. Feng M. G. Ying S. H. (2019). Autophagy-related gene ATG7 participates in the asexual development, stress response and virulence of filamentous insect pathogenic fungus *Beauveria bassiana*. Curr. Genet. 65, 1015–1024. 10.1007/s00294-019-00955-1 30879087

[B46] Loeb J. D. Sepulveda-Becerra M. Hazan I. Liu H. (1999). A G1 cyclin is necessary for maintenance of filamentous growth in *Candida albicans*. Mol. Cell. Biol. 19, 4019–4027. 10.1128/MCB.19.6.4019 10330142 PMC104361

[B47] Luginbuehl L. H. Oldroyd G. E. D. (2017). Understanding the arbuscule at the heart of endomycorrhizal symbioses in plants. Curr. Biol. 27, R952–R963. 10.1016/j.cub.2017.06.042 28898668

[B48] Machuca A. Pereira G. Aguiar A. Milagres A. M. F. (2007). Metal-chelating compounds produced by ectomycorrhizal fungi collected from pine plantations. Lett. Appl. Microbiol. 44, 7–12. 10.1111/j.1472-765X.2006.02046.x 17209807

[B49] Macias-Benitez S. Garcia-Martinez A. M. Jimenez P. C. Gonzalez J. M. Moral M. T. Rubio J. P. (2020). Rhizospheric organic acids as biostimulants: monitoring feedbacks on soil microorganisms and biochemical properties. Front. Plant Sci. 11:633. 10.3389/fpls.2020.00633 32547578 PMC7270406

[B50] Maclean A. M. Bravo A. Harrison M. J. (2017). Plant signaling and metabolic pathways enabling arbuscular mycorrhizal symbiosis. Plant Cell 29, 2319–2335. 10.1105/tpc.17.00555 28855333 PMC5940448

[B51] Maillet F. Poinsot V. Andre O. Puech-Pages V. Haouy A. Gueunier M.. (2011). Fungal lipochitooligosaccharide symbiotic signals in arbuscular mycorrhiza. Nature 469, 58–U1501. 10.1038/nature09622 21209659

[B52] Malkov N. Fliegmann J. Rosenberg C. Gasciolli V. Timmers A. C. J. Nurisso A.. (2016). Molecular basis of lipo-chitooligosaccharide recognition by the lysin motif receptor-like kinase LYR3 in legumes. Biochem. J. 473, 1369–1378. 10.1042/BCJ20160073 26987814

[B53] Marburger D. A. Willbur J. F. Weber M. E. Ane J. M. Kabbage M. Conley S. P.. (2018). Characterizing the effect of foliar lipo-chitooligosaccharide application on sudden death syndrome and sclerotinia stem rot in soybean. Plant Health Prog. 19, 46–53. 10.1094/PHP-10-17-0058-RS30812563

[B54] Martin F. Aerts A. Ahrén D. Brun A. Danchin E. G. J. Duchaussoy F.. (2008). The genome of Laccaria bicolor provides insights into mycorrhizal symbiosis. Nature 452, 88–92. 10.1038/nature06556 18322534

[B55] Martin F. Selosse M. A. (2008). The *Laccaria* genome: a symbiont blueprint decoded. New Phytol. 180, 296–310. 10.1111/j.1469-8137.2008.02613.x 19138220

[B56] Mukherjee A. Ane J. M. (2011). Germinating spore exudates from arbuscular mycorrhizal fungi: molecular and developmental responses in plants and their regulation by ethylene. Mol. Plant-Microbe Interact. 24, 260–270. 10.1094/MPMI-06-10-0146 21043574

[B57] Pan Y. D. Birdsey R. A. Phillips O. L. Jackson R. B. (2013). The Structure, distribution, and biomass of the world's forests. Ann. Rev. Ecol. Evolut. System. 44, 593–622. 10.1146/annurev-ecolsys-110512-135914

[B58] Park H. O. Bi E. (2007). Central roles of small GTPases in the development of cell polarity in yeast and beyond. Microbiol. Mol. Biol. Rev. 71, 48–96. 10.1128/MMBR.00028-06 17347519 PMC1847380

[B59] Pedraza N. Cemeli T. Monserrat M. V. Gari E. Ferrezuelo F. (2019). Regulation of small GTPase activity by G1 cyclins. Small GTPases 10, 47–53. 10.1080/21541248.2016.1268665 28129038 PMC6343608

[B60] Pellegrin C. Daguerre Y. Ruytinx J. Guinet F. Kemppainen M. Frey N. F. D.. (2019). *Laccaria bicolor* MiSSP8 is a small-secreted protein decisive for the establishment of the ectomycorrhizal symbiosis. Environ. Microbiol. 21, 3765–3779. 10.1111/1462-2920.14727 31260142

[B61] Plassard C. Fransson P. (2009). Regulation of low-molecular weight organic acid production in fungi. Fungal Biol. Rev. 23, 30–39. 10.1016/j.fbr.2009.08.002

[B62] Plett J. M. Daguerre Y. Wittulsky S. Vayssieres A. Deveau A. Melton S. J.. (2014). Effector MiSSP7 of the mutualistic fungus *Laccaria bicolor* stabilizes the *Populus* JAZ6 protein and represses jasmonic acid (JA) responsive genes. Proc. Natl. Acad. Sci. U.S.A. 111, 8299–8304. 10.1073/pnas.1322671111 24847068 PMC4050555

[B63] Plett J. M. Martin F. M. (2018). Know your enemy, embrace your friend: using omics to understand how plants respond differently to pathogenic and mutualistic microorganisms. Plant J. 93, 729–746. 10.1111/tpj.13802 29265527

[B64] Poinsot V. Crook M. B. Erdn S. Maillet F. Bascaules A. Ane J. M. (2016). New insights into Nod factor biosynthesis: Analyses of chitooligomers and lipo-chitooligomers of *Rhizobium* sp. IRBG74 mutants. Carbohydrate Res. 434, 83-93. 10.1016/j.carres.2016.08.001 27623438 PMC5080398

[B65] Pollack J. K. Harris S. D. Marten M. R. (2009). Autophagy in filamentous fungi. Fungal Genet. Biol. 46, 1–8. 10.1016/j.fgb.2008.10.010 19010432

[B66] Polpitiya A. D. Qian W. J. Jaitly N. Petyuk V. A. Adkins J. N. Camp D. G. 2nd Anderson G. A.. (2008). DAnTE: a statistical tool for quantitative analysis of -omics data. Bioinformatics 24, 1556–1558. 10.1093/bioinformatics/btn217 18453552 PMC2692489

[B67] Prinz S. Avila-Campillo I. Aldridge C. Srinivasan A. Dimitrov K. Siegel A. F.. (2004). Control of yeast filamentous-form growth by modules in an integrated molecular network. Genome Res. 14, 380–390. 10.1101/gr.2020604 14993204 PMC353223

[B68] Raina M. Ibba M. (2014). TRNAs as regulators of biological processes. Front. Genet. 5:171. 10.3389/fgene.2014.00171 24966867 PMC4052509

[B69] Ranocchi B. Amicucci A. (2021). GTPases in hyphal growth, in Encyclopedia of Mycology, ed. Zaragoza C. A. (Amsterdam: Elsevier), 32–43. 10.1016/B978-0-12-819990-9.00050-0

[B70] Reich M. Gobel C. Kohler A. Buee M. Martin F. Feussner I.. (2009). Fatty acid metabolism in the ectomycorrhizal fungus *Laccaria bicolor*. New Phytol. 182, 950–964. 10.1111/j.1469-8137.2009.02819.x 19383096

[B71] Riquelme M. Roberson R. W. Mcdaniel D. P. Bartnicki-Garcia S. (2002). The effects of ropy-1 mutation on cytoplasmic organization and intracellular motility in mature hyphae of *Neurospora crassa*. Fungal Genet. Biol. 37, 171–179. 10.1016/S1087-1845(02)00506-6 12409101

[B72] Rodriguez-Morgado B. Jimenez P. C. Moral M. T. Rubio J. P. (2017). Effect of L-lactic acid from whey wastes on enzyme activities and bacterial diversity of soil. Biol. Fertil. Soils 53, 389–396. 10.1007/s00374-017-1187-z

[B73] Rua D. Tobe B. T. Kron S. J. (2001). Cell cycle control of yeast filamentous growth. Curr. Opin. Microbiol. 4, 720–727. 10.1016/S1369-5274(01)00274-0 11731325

[B74] Rush T. A. Puech-Pages V. Bascaules A. Jargeat P. Maillet F. Haouy A.. (2020). Lipo-chitooligosaccharides as regulatory signals of fungal growth and development. Nat. Commun. 11:3897. 10.1038/s41467-020-17615-5 32753587 PMC7403392

[B75] Seiler S. Plamann M. Schliwa M. (1999). Kinesin and dynein mutants provide novel insights into the roles of vesicle traffic during cell morphogenesis in *Neurospora*. Curr. Biol. 9, 779–785. 10.1016/S0960-9822(99)80360-1 10469561

[B76] Shannon P. Markiel A. Ozier O. Baliga N. S. Wang J. T. Ramage D.. (2003). Cytoscape: a software environment for integrated models of biomolecular interaction networks. Genome Res. 13, 2498–2504. 10.1101/gr.1239303 14597658 PMC403769

[B77] Siczek A. Lipiec J. Wielbo J. Kidaj D. Szarlip P. (2014). Symbiotic activity of pea (*Pisum sativum*) after application of nod factors under field conditions. Int. J. Mol. Sci. 15, 7344–7351. 10.3390/ijms15057344 24786094 PMC4057676

[B78] Skiada V. Avramidou M. Bonfante P. Genre A. Papadopoulou K. K. (2020). An endophytic *Fusarium*–legume association is partially dependent on the common symbiotic signalling pathway. New Phytol. 226, 1429–1444. 10.1111/nph.16457 31997356

[B79] Soanes D. M. Alam I. Cornell M. Wong H. M. Hedeler C. Paton N. W.. (2008). Comparative genome analysis of filamentous fungi reveals gene family expansions associated with fungal pathogenesis. PLoS ONE 3:e2300. 10.1371/journal.pone.0002300 18523684 PMC2409186

[B80] Sommer R. Makshakova O. N. Wohlschlager T. Hutin S. Marsh M. Titz A.. (2018). Crystal structures of fungal tectonin in complex with O-Methylated Glycans suggest key role in innate immune defense. Structure 26, 391–402 e394. 10.1016/j.str.2018.01.003 29398527

[B81] Sun J. H. Miller J. B. Granqvist E. Wiley-Kalil A. Gobbato E. Maillet F.. (2015). Activation of symbiosis signaling by arbuscular mycorrhizal fungi in legumes and rice. Plant Cell 27, 823–838. 10.1105/tpc.114.131326 25724637 PMC4558648

[B82] Sun L. Dong S. Ge Y. Fonseca J. P. Robinson Z. T. Mysore K. S.. (2019). DiVenn: an interactive and integrated web-based visualization tool for comparing gene lists. Front. Genet. 10:421. 10.3389/fgene.2019.00421 31130993 PMC6509638

[B83] Tanaka K. Cho S. H. Lee H. Pham A. Q. Batek J. M. Cui S.. (2015). Effect of lipochitooligosaccharide on early growth of C4 grass seedlings. J. Exp. Bot. 66, 5727–5738. 10.1093/jxb/erv260 26049159 PMC4566972

[B84] Tedersoo L. Bahram M. Zobel M. (2020). How mycorrhizal associations drive plantpopulation and community biology. Science 367:eaba1223. 10.1126/science.aba1223 32079744

[B85] Truesdell G. M. Jones C. Holt T. Henderson G. Dickman M. B. (1999). A Ras protein from a phytopathogenic fungus causes defects in hyphal growth polarity, and induces tumors in mice. Mol. General Genet. 262, 46–54. 10.1007/s004380051058 10503535

[B86] Tschaplinski T. J. Plett J. M. Engle N. L. Deveau A. Cushman K. C. Martin M. Z.. (2014). *Populus trichocarpa* and *Populus deltoides* exhibit different metabolomic responses to colonization by the symbiotic fungus *Laccaria bicolor*. Mol. Plant Microbe Interact. 27, 546–556. 10.1094/MPMI-09-13-0286-R 24548064

[B87] Tyanova S. Temu T. Sinitcyn P. Carlson A. Hein M. Y. Geiger T.. (2016). The Perseus computational platform for comprehensive analysis of (prote)omics data. Nat. Methods 13, 731–740. 10.1038/nmeth.3901 27348712

[B88] Van Munster J. M. Nitsche B. M. Akeroyd M. Dijkhuizen L. Van Der Maarel M. J. E. C. Ram A. F. J. (2015). Systems approaches to predict the functions of glycoside hydrolases during the life cycle of *Aspergillus niger* using developmental mutants delta brlA and Delta flbA. PLoS ONE 10:0116269. 10.1371/journal.pone.0116269 25629352 PMC4309609

[B89] Wessels J. G. H. (1990). Role of cell wall architecture in fungal tip growth generation, in Tip Growth In Plant and Fungal Cells, ed. Heath I. B. (Cambridge, MA: Academic Press), 1–29. 10.1016/B978-0-12-335845-5.50004-5

[B90] White S. Mcintyre M. Berry D. R. Mcneil B. (2002). The autolysis of industrial filamentous fungi. Crit. Rev. Biotechnol. 22, 1–14. 10.1080/07388550290789432 11958333

[B91] Wohlschlager T. Butschi A. Grassi P. Sutov G. Gauss R. Hauck D.. (2014). Methylated glycans as conserved targets of animal and fungal innate defense. Proc. Natl. Acad. Sci. U.S.A. 111, E2787–E2796. 10.1073/pnas.1401176111 24879441 PMC4103367

[B92] Wu C. Qu J. Liu L. Kang H. Sun H. Zhang Y.. (2021). Quo vadis: signaling molecules and small secreted proteins from mycorrhizal fungi at the early stage of mycorrhiza formation. Symbiosis 8, 123–143. 10.1007/s13199-021-00793-1

[B93] Xiang X. Fischer R. (2004). Nuclear migration and positioning in filamentous fungi. Fungal Genet. Biol. 41, 411–419. 10.1016/j.fgb.2003.11.010 14998524

[B94] Yamamoto A. Hiraoka Y. (2003). Cytoplasmic dynein in fungi: insights from nuclear migration. J. Cell Sci. 116, 4501–4512. 10.1242/jcs.00835 14576344

[B95] Yin H. Du Y. Dong Z. (2016). Chitin oligosaccharide and chitosan oligosaccharide: two similar but different plant elicitors. Front. Plant Sci. 7:522. 10.3389/fpls.2016.00522 27148339 PMC4840203

