# Supplementary figures and images for: Expanding the Biological Role of Lipo-Chitooligosaccharides and Chitooligosaccharides in Laccaria bicolor Growth and Development
Source: Front Fungal Biol. 2022 Feb 14;3:808578. doi: 10.3389/ffunb.2022.808578 (PMC10512320; doi:10.3389/ffunb.2022.808578)

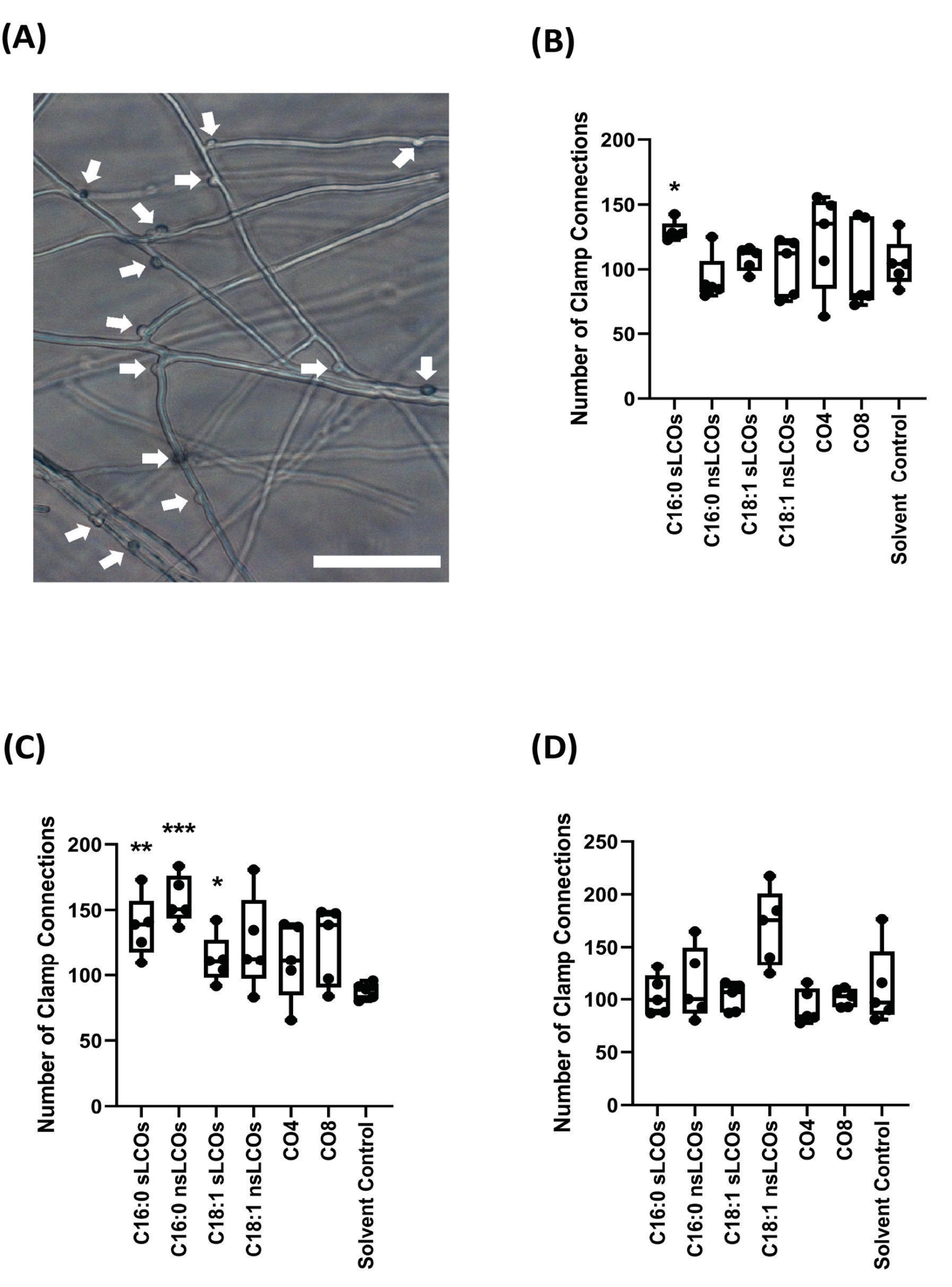

Supplement: Supplementary file 1 [file Image_1.TIFF]

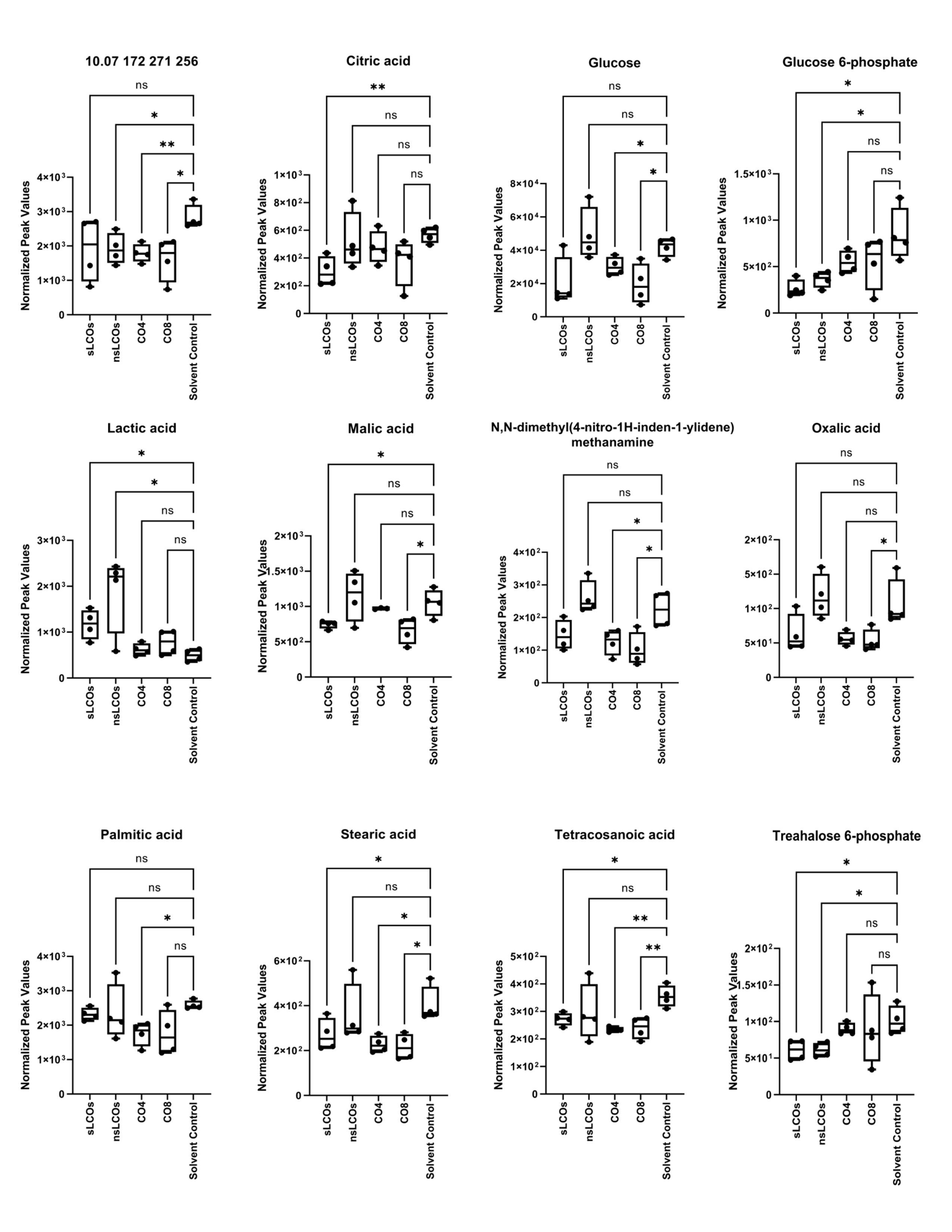

Supplement: Supplementary file 2 [file Image_2.TIFF]

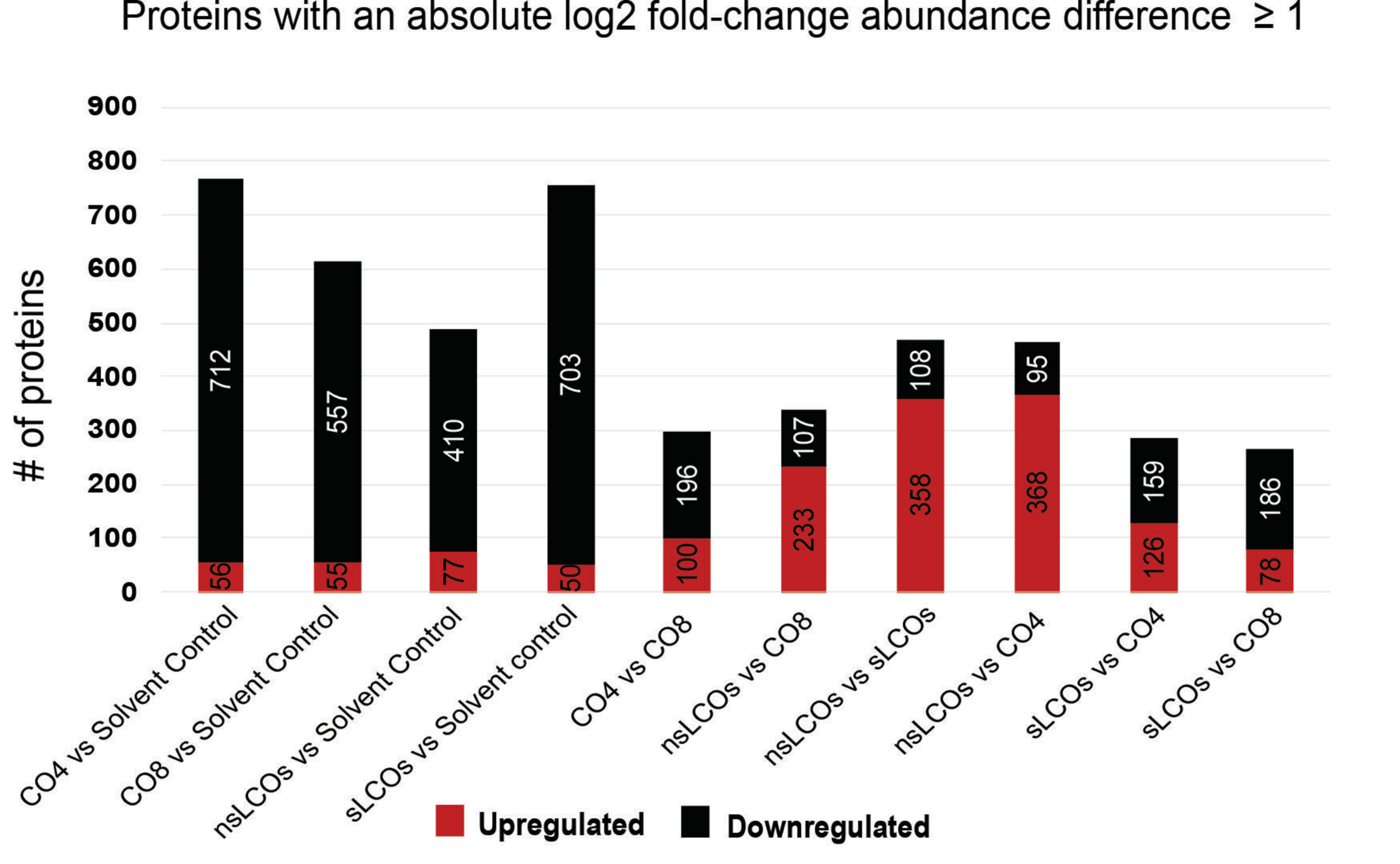

Supplement: Supplementary file 3 [file Image_3.TIFF]
