# Supplementary material for: Expanding the Biological Role of Lipo-Chitooligosaccharides and Chitooligosaccharides in Laccaria bicolor Growth and Development
Source: Front Fungal Biol. 2022 Feb 14;3:808578. doi: 10.3389/ffunb.2022.808578 (PMC10512320; doi:10.3389/ffunb.2022.808578)
Supplement: Supplementary file 8 [file Data_Sheet_1.DOCX]

**Captions for Supplementary Materials**

**Figure S1. LCOs and COs effect on clamp connections.** (A) Clamp connections (white arrows) observed under the microscope in *L. bicolor* mycelia. Boxplots showing the number of clamp connections counted over (B) 7 days post-inoculation (dpi), (C) 15 dpi, and (D) 21 dpi. Welch’s ANOVA test was significant for days 7 (*p­*-value = 0.0186), and 15 (*p­*-value = 0.0002). Treatments were compared to the solvent control with Welch’s unpaired t-test. N is five biological replications with five technical replications each.

**Figure S2. Metabolites with significant abundance changes.** Metabolite abundance changes in treatments were compared to solvent control with Welch’s unpaired t-test. Welch’s ANOVA p-value <0.05. Stearic acid (p-value = 0.0533) and oxalic acid (p-value = 0.0504) are also included in the figure. (*) p-value <0.05; (**) p-value <0.01; (***) p-value <0.001. N is four biological replications. Treatment CO4 for the malic acid analysis had an outlier removed.

**Figure S3. Distribution of up and downregulated proteins in the comparisons of *L. bicolor* COs and LCOs-treated samples to solvent controls.** Proteins plotted in the stacked bar plots were significant by ANOVA test (p-value < 0.05) and had an absolute log2 fold change ≥1 in at least one significant treatment pair.

**Table S1. Abundance values and ANOVA -** Tukey's HSD statistical analysis of all quantifiable proteins identified via LC-MS/MS from *L. bicolor* COs and LCOs-treated samples.

**Table S2. [Sheet1] List of downregulated proteins identified in common between all COs and LCOs treated samples compared to solvent control. [Sheet 2] List of downregulated proteins identified between CO4, CO8, and sLCOs-treated samples compared to solvent control samples. Also shown, the GO term enrichment results performed for these proteins.**

**Table S3.** **List of unique upregulated proteins between comparisons of *L. bicolor* treated samples versus solvent controls.** A table of predicted, hypothetical and unknown function proteins and their sequence lengths is also provided alongside their predicted signal peptides by SignalP v5.0

**Table S4. STRING Network interaction data from all downregulated proteins belonging to the "cellular component assembly" and "establishment of cell polarity" enriched GO categories.** Disconnected nodes in the network are not reported.
